# Diagnostic Accuracy of Mental Health Screening Tools After Mild Traumatic Brain Injury

**DOI:** 10.1001/jamanetworkopen.2024.24076

**Published:** 2024-07-23

**Authors:** Michelle Gitaari, Ana Mikolić, William J. Panenka, Noah D. Silverberg

**Affiliations:** 1 Department of Psychology, University of British Columbia, Vancouver, Canada; 2 Rehabilitation Research Program, Vancouver Coastal Health Research Institute, Vancouver, British Columbia, Canada; 3 BC Mental Health and Substance Use Research Institute, Burnaby, British Columbia, Canada; 4 British Columbia Provincial Neuropsychiatry Program, Vancouver, British Columbia, Canada; 5 Department of Psychiatry, University of British Columbia, Vancouver, Canada; 6 Djavad Mowafaghian Centre for Brain Health, Vancouver, British Columbia, Canada

## Abstract

**Importance:**

Mental health disorders are common after mild traumatic brain injury (mTBI) and likely exacerbate postconcussive symptoms and disability. Early detection could improve clinical outcomes, but the accuracy of mental health screening tools in this population has not been well established.

**Objective:**

To determine the diagnostic accuracy of the Patient Health Questionnaire–9 (PHQ-9), Generalizaed Anxiety Disorder–7 (GAD-7), and Primary Care PTSD (Posttramatic Stress Disorder) Screen for *Diagnostic and Statistical Manual of Mental Disorders* (Fifth Edition) (*DSM-5*) (PC-PTSD-5) in adults with mTBI.

**Design, Setting, and Participants:**

This diagnostic study was performed as a secondary analysis of a cluster randomized clinical trial. Self-report mental health screening tools (PHQ-9, GAD-7, and PC-PTSD-5) were administered online 12 weeks after mTBI and compared against a structured psychodiagnostic interview (Mini-International Neuropsychiatric Interview for *DSM-5* (MINI) over videoconference at the same time. Adults with mTBI (N = 537) were recruited from February 1, 2021, to October 25, 2022.

**Main Outcomes and Measures:**

Presence of a major depressive episode, anxiety disorders, and PTSD were determined by a blinded assessor with the MINI. Diagnostic accuracy statistics were derived for the PHQ-9, GAD-7, and PC-PTSD-5. Findings were disaggregated for participants with and without persistent postconcussion symptoms (PPCS) by *International and Statistical Classification of Diseases, Tenth Revision* criteria.

**Results:**

Data were available for 499 of 537 trial participants, 278 (55.7%) of whom were female; the mean (SD) age was 38.8 (13.9) years. Each screening questionnaire had strong diagnostic accuracy in the overall sample for optimal cut points (area under the curve [AUC], ≥0.80; sensitivity, 0.55-0.94; specificity, 0.64-0.94). The AUC (difference of 0.01-0.13) and specificity (difference, 5-65 percentage points) were lower in those with PPCS present compared with PPCS absent, but the prevalence of at least 1 mental health disorder was 3 to 5 times higher in patients with PPCS present. The GAD-7 had slightly better performance than the PC-PTSD-5 for detecting PTSD (AUC, 0.85 [95% CI, 0.80-0.89] vs 0.80 [95% CI, 0.72-0.87]). The optimal cutoff on the PHQ-9 was 5 or more symptoms experienced on more than half of days; on the GAD-7, a total score of at least 7.

**Conclusions and Relevance:**

The findings of this diagnostic study suggest that the PHQ-9, GAD-7 and PC-PTSD-5 accurately screen for mental health disorders in patients with mTBI. Future research should corroborate optimal test cutoffs for this population.

## Key Points

**Question:**

Can self-report screening tools accurately detect a major depressive episode, anxiety disorder, and posttraumatic stress disorder (PTSD) after mild traumatic brain injury (mTBI)?

**Findings:**

In this diagnostic study with 499 participants with mTBI, the Patient Health Questionnaire–9, Generalized Anxiety Disorder–7, and Primary Care PTSD Screen for the *Diagnostic and Statistical Manual of Mental Disorders* (Fifth Edition) screening tools had acceptable diagnostic accuracy; the Generalized Anxiety Disorder–7 accurately identified not only anxiety disorders but also PTSD. In patients with persistent postconcussive symptoms, specificity was lower and mental health disorders were more common.

**Meaning:**

These findings suggest that brief self-report tools can reliably screen for mental health disorders after mTBI.

## Teaser

This diagnostic study evaluates the accuracy of self-report screening tools for major depressive episodes, anxiety disorders, and posttraumatic stress disorder compared with a structured diagnostic interview among patients with mild traumatic brain injury (TBI).

## Introduction

Individuals with mild traumatic brain injury (mTBI) are more likely to develop mental health conditions such as depression, anxiety disorders, and posttraumatic stress disorder (PTSD) compared with the general population and those with orthopedic injuries.^1,2,3,4,5^ The prevalence rates for mental health conditions 3 to 12 months after mTBI are 17% to 27% for depressive disorders,^1,6,7^ 11% to 24% for anxiety disorders,^6,8,9^ and 10% to 21% for PTSD.^3,5,10,11^ Mental health disorders likely exacerbate persistent postconcussion symptoms (PPCS)^11,12,13,14,15^ and contribute to the substantial rate (30%-50%) of chronic disability after mTBI.^12,16^ Outcomes from mTBI could be optimized by proactively monitoring for new or worsened mental health disorders (eg, with self-report screening scales) and initiating mental health treatment.^15^

Mental health disorders can be efficiently detected in primary care with self-report screening tools such as the Patient Health Questionnaire–9 (PHQ-9) for a current major depressive episode (MDE),^17,18^ General Anxiety Disorder–7 (GAD-7) for anxiety disorders,^19^ and Primary Care PTSD Screen for the *DSM-5* (*Diagnostic and Statistical Manual of Mental Disorders* [Fifth Edition]) (PC-PTSD-5) for PTSD.^20,21^ The PHQ-9 and GAD-7 are among the most extensively studied and widely used mental health screens in primary care. Meta-analyses have demonstrated good diagnostic accuracy for the PHQ-9 in identifying MDE, with no substantial differences between investigated subgroups (eg, sex and recruitment setting),^22,23,24^ and that the GAD-7 can detect generalized anxiety disorder and other anxiety disorders.^25^ Preliminary evidence suggests that these tools have comparable diagnostic accuracy in mTBI, at least in outpatient^26,27,28^ and hospitalized samples with trauma-related intracranial lesions,^29^ but establishing diagnostic accuracy in larger and more generalizable samples with mTBI is needed (only 15% of adults with mTBI have trauma-related intracranial lesions).^30^ It is also unclear whether optimal cutoff scores differ for patients with mTBI, especially in the presence of PPCS. Sleep disturbance, fatigue, concentration difficulties, and mood symptoms are transdiagnostic, that is, common to both PPCS and mental health disorders,^14,31^ raising concerns about the performance of self-report screening tools in mTBI.

The PC-PTSD-5 was originally developed for use with military service members and veterans.^32^ It appears to be useful in primary care^20^ but has not been evaluated as extensively as the PHQ-9 or GAD-7, nor has it been previously studied in mTBI. Its appeal in this population is that it only queries core PTSD symptoms that are specific to PTSD (ie, those that do not overlap with PPCS). Evidence from primary care studies suggests that the GAD-7 may be sensitive to PTSD,^25,33,34,35,36^ but this has not been evaluated within the context of mTBI.

Clinical practice guidelines for mTBI recommend routine screening for mental health disorders,^37,38^ but insufficient evidence is available to guide implementation of this recommendation. The present study evaluated the diagnostic accuracy of the PHQ-9, GAD-7, and PC-PTSD-5 screening tools against a criterion standard structured diagnostic interview identification of MDE, anxiety disorders, and PTSD. Secondary aims were to compare diagnostic accuracy in those with and without PPCS, to determine whether the GAD-7 can simultaneously screen for PTSD as well as a PTSD-specific screening tool (the PC-PTSD-5), and to determine whether the combination of the GAD-7 and PC-PTSD-5 can optimize PTSD screening.

## Methods

This diagnostic study was a secondary analysis of a cluster randomized clinical trial that analyzed whether a clinical practice guideline implementation tool designed to support early detection of mental health disorders after mTBI could lower the risks of mental health complications.^39,40^ The study was reviewed and approved by the University of British Columbia Clinical Research Ethics Board, and all participants gave informed consent to participate. We followed the Standards for Reporting of Diagnostic Accuracy (STARD) reporting guidelines.

### Participants

Participants were recruited from February 1, 2021, to October 15, 2022. A total of 537 participants with an mTBI were recruited from 6 emergency departments and 2 urgent care centers in the Greater Vancouver area in British Columbia, Canada. Participants were included if they were aged 18 to 69 years, presented to care within 72 hours, met World Health Organization Neurotrauma Task Force criteria for mTBI (eMethods in Supplement 1),^41^ were fluent in English, had their primary residence in British Columbia, and designated a general practitioner or a walk-in clinic where they would seek follow-up care. Individuals with preexisting unstable or serious illnesses were excluded.

Participant race and ethnicity were self-reported and included the following categories: Arab; Black; Caribbean; East or Southeast Asian; Fijian; First Nation, Inuit, or Métis; Latinx; Middle Eastern; South Asian; White; and preferred not to answer. These data were collected to provide information about the generalizability of the study results.

Participants consented by completing an online form through REDCap (Research Electronic Data Capture; Vanderbilt University).^42^ Details about the recruitment procedures are available in the trial protocol paper.^39^

### Measures

#### Patient Health Questionnaire–9

The PHQ-9^17^ is a 9-question assessment developed to measure depressive symptoms. Participants rate how frequently they experienced symptoms in the past 2 weeks, from 0 (not at all) to 3 (nearly every day). The total score ranges from 0 to 27. A range of cutoffs have been proposed, with total score of 10 or more being most common. Additionally, 3 algorithms have been considered for identifying MDEs that require the endorsement of at least 1 cardinal symptom (depression or anhedonia) and 1 of the following: a score of at least 10,^28,29^ 5 or more symptoms rated as experienced more than half of the days (suicidal ideation is counted if endorsed with any frequency),^17,29^ and 5 or more symptoms rated as experienced several days (eTable 1 in Supplement 1).^29^

#### Generalized Anxiety Disorder–7 

The GAD-7 is a 7-question screening tool that measures anxiety symptoms.^19^ Participants rate how frequently they have experienced symptoms over the past 2 weeks from 0 (not at all) to 3 (nearly every day). The total scores range from 0 to 21. The cutoff scores of at least 7^27^ and at least 10^19^ have been proposed for the indication of an anxiety disorder (eTable 1 in Supplement 1). In addition to these accepted cutoff scores, we also investigated cutoff scores ranging from 5 to 15 (eTables 10, 12, and 14 in Supplement 1). The diagnostic accuracy of the GAD-7 for generalized anxiety disorder is provided in eTables 16 to 21 in Supplement 1.

#### Primary Care PTSD Screen for *DSM-5*

The PC-PTSD-5 is a 5-question instrument.^32^ First, participants are asked about their lifetime trauma exposure. If they indicate that they have had no exposure to an unusual, frightening, traumatic, or horrific event, their score is 0. If they have been exposed to such an event, participants respond 0 (indicating no) or 1 (indicating yes) to 5 questions about symptoms they may be experiencing, with the total score ranging from 0 to 5. Cutoff scores of at least 3^21,32^ and at least 4^20^ have been proposed for indication of PTSD (eTable 1 in Supplement 1).

#### Rivermead Post-Concussion Questionnaire

The Rivermead Post-Concussion Symptoms Questionnaire (RPQ) consists of 16 symptoms. Each symptom is rated on a scale from 0 (indicating not experienced at all) to 4 (indicating a severe problem).^43^

#### Mini-International Neuropsychiatric Interview

The MINI is a structured diagnostic interview that is designed to assess the most common psychiatric disorders in clinical and research settings.^44^ We administered 8 modules assessing MDEs, anxiety disorders (ie, panic disorder, agoraphobia, social anxiety disorder, specific phobia, generalized anxiety disorder, or obsessive-compulsive disorder), and PTSD. Version 7.0.2 of the MINI is designed to align with *DSM-5*.^31^

### Procedure

At 12 weeks post injury, outcome assessors (ie, graduate students of clinical psychology or rehabilitation science) administered the MINI through a video-conferencing platform under the supervision of a registered psychologist (N.D.S.) to eligible participants. The outcome assessors were certified in Adult Standard MINI 7.0.2 Training provided by the publisher and participated in weekly group supervision with the psychologist to discuss complex cases and resolve uncertain MINI coding. To ensure standardized administration, assessors were periodically audited (by the supervising psychologist observing an assessment live) to prevent drift from standardized administration.

After administering the MINI, participants were e-mailed a REDCap^42^ online survey that included the PHQ-9, GAD-7, PC-PTSD-5, and the RPQ. The outcome assessors were blind to the results of the screening questionnaires.

### Statistical Analysis

Participant characteristics and responses on the screening questionnaires were described in terms of central tendency. Missing responses on the screening tools were replaced with the mean score of the questionnaire if participants missed fewer than 20% of items (26 participants [5.2%] missed 1 to 3 responses on the RPQ, 19 [3.8%] missed 1 response on the PHQ-9, 8 [1.6%] missed 1 response on the GAD-7, and 3 [0.6%] missed 1 response on the PC-PTSD-5).

We derived the receiver operating characteristics curve to determine the area under the curve (AUC) of the total scores of each screening questionnaire for the diagnosis of MDE (based on meeting criteria for a current MDE), any anxiety disorder (≥1), or PTSD according to the results of the MINI. An AUC of 1.0 describes perfect discrimination.^45^ Additionally, we investigated the sensitivity, specificity, positive predictive values (PPV), negative predictive values (NPV), and likelihood ratios at various cutoff scores. Finally, we determined 95% CIs by using the exact binomial confidence interval method.^46^

We assessed the diagnostic accuracy in the overall sample and separately for those with and without PPCS. Patients were categorized as having PPCS present or absent based on their endorsement of symptoms on the RPQ that met the *International and Statistical Classification of Diseases, Tenth Revision*, category C criteria for postconcussional syndrome (F07.81), as in previous studies.^47,48^ The RPQ assesses for categories I through IV (physical, emotional, and cognitive symptoms and poor sleep) of postconcussional syndrome, and participants were categorized as having PPCS present if they endorsed at least 1 symptom as moderate to severe (ie, item scoring ≥3) in at least 3 categories.^49^

To determine whether the diagnostic accuracy of the GAD-7 is equivalent to the PC-PTSD-5 when detecting symptoms of PTSD, we compared their AUCs, sensitivity, and specificity at various cutoffs. Additionally, we performed a multivariable logistic regression to explore whether combining the questionnaires’ total scores would improve the ability to detect PTSD, measured by using likelihood ratio tests.

The analyses were performed using R packages pROC, version 1.18.0^50^; rms, version 6.8-1^51^; and dplyr, version 1.1.4^52^ (R Project for Statistical Computing), and the DAG_STAT spreadsheet (Andew J. Mackinnon).^53^ Comparisons were made using a 1-tailed *t* test; statistical significance was set at *P* < .05 (eTables 2-31 in Supplement 1).

## Results

### Participants

The final sample included 499 participants (278 [55.7%] female; 221 [44.3%] male; mean [SD] age, 38.8 [13.9] years) who completed the MINI, PHQ-9, GAD-7, and PC-PTSD-5 at 12 weeks (eFigure in Supplement 1). In terms of race and ethnicity, 1 participant (0.2%) was Arab; 7 (1.4%) were Black; 2 (0.4%) were Caribbean; 103 (20.6%) were East or Southeast Asian; 1 (0.2%) was Fijian; 12 (2.4%) were First Nation, Inuit, or Métis; 11 (2.2%) were Latinx; 21 (4.2%) were Middle Eastern; 30 (6.0%) were South Asian; 324 (64.9%) were White; and 10 (2.0%) preferred not to answer. Participant characteristics are summarized in Table 1.

**Table 1.** Sociodemographic and Injury Characteristics of Participants

Characteristics	Participants (N = 499)^a^
Age, mean (SD) [range], y	38.8 (13.9) [18-69]
Sex	
Male	221 (44.3)
Female	278 (55.7)
Gender	
Men	222 (44.5)
Women	271 (54.3)
Other	6 (1.2)
Race and ethnicity^b^	
Arab	1 (0.2)
Black	7 (1.4)
Caribbean	2 (0.4)
East or Southeast Asian	103 (20.6)
Fijian	1 (0.2)
First Nation, Inuit, or Métis	12 (2.4)
Latinx	11 (2.2)
Middle Eastern	21 (4.2)
South Asian	30 (6.0)
White	324 (64.9)
Prefer not to answer	10 (2.0)
Educational level^c^	
Less than grade 12	7 (1.4)
High school graduate	92 (18.4)
Some college	49 (9.8)
2-y College degree	89 (17.8)
Bachelor’s degree	178 (35.7)
Graduate or professional degree	83 (16.6)
Prior mental health disorder and treatment^b^	
Depression	159 (31.9)
Anxiety	167 (33.5)
Treatment	367 (73.5)
Cause of injury	
Motor vehicle accident	112 (22.4)
Assault	38 (7.6)
Fall	171 (34.3)
Sport or recreation accident	111 (22.2)
Other injury	67 (13.4)
Loss of consciousness	
No	258 (51.7)
Yes	190 (38.1)
Unknown	50 (10.0)
GCS score at hospital	
15	403 (80.8)
14	27 (5.4)
13	1 (0.2)
Not documented	68 (13.6)
Trauma-related abnormalities on head computed tomography	
Present	18 (3.6)
Absent	248 (49.7)
Head computed tomography not ordered	233 (46.7)

Abbreviation: GCS, Glasgow Coma Scale.

^a^ Unless otherwise indicated, data are expressed as No. (%) of participants. Percentages have been rounded and may not total 100.

^b^ Participants were able to select more than 1 response.

^c^ One participant (0.2%) did not report educational level.

### Prevalence of Mental Health Disorders

On the MINI, one-third of patients met criteria for at least 1 mental health disorder: 140 (28.1%) for at least 1 anxiety disorder, 102 (20.4%) for MDE, and 48 (9.6%) for PTSD (Table 2). The prevalence of at least 1 mental health disorder was greater in participants with PPCS present (117 of 158 [74.1%]) compared with participants with PPCS absent (68 of 341 [19.9%]; relative risk [RR], 3.71 [95% CI, 2.95-4.68]), including MDE (RR, 10.97 [95% CI, 6.64-17.53]), at least 1 anxiety disorder (RR, 2.96 [95% CI, 2.25-3.91]), and PTSD (RR, 9.35 [95% CI, 4.65-18.83]).

**Table 2.** Prevalence of Disorders and Distributions of Screening Questionnaires

Measure	Participant group		
	Overall (N = 499)	PPCS present (n = 158)	PPCS absent (n = 341)
MINI, No. (%)			
Major depressive episode	102 (20.4)	85 (53.8)	17 (5.0)
Any anxiety disorder	140 (28.1)	81 (51.3)	59 (17.3)
PTSD	48 (9.6)	39 (24.7)	9 (2.6)
PHQ-9 score, mean (SD) [range]^a^	7.3 (6.5) [0-27]	14.1 (5.6) [2-27]	4.2 (4.1) [0-22]
GAD-7 score, mean (SD) [range]^a^	5.8 (5.7) [0-21]	11.1 (5.4) [1-21]	3.4 (3.9) [0-21]
PC-PTSD-5 score, mean (SD) [range]^b^	1.0 (1.6) [0-5]	1.8 (1.9) [0-5]	0.6 (1.2) [0-5]
RPQ score, mean (SD) [range]^c^	20.6 (13.6) [0-58]	35.9 (8.5) [16-58]	13.6 (9.0) [0-39]

Abbreviations: GAD-7, Generalized Anxiety Disorder–7; MINI, Mini-International Neuropsychiatric Interview; PC-PTSD-5, Primary Care PTSD (Posttraumatic Stress Disorder) Screen for *DSM-5* (*Diagnostic and Statistical Manual of Mental Disorders* [Fifth Edition]); PHQ-9, Patient Health Questionnaire–9; PPCS, persistent postconcussive symptoms; RPQ, Rivermead Post-Concussion Symptoms Questionnaire.

^a^ Higher scores indicate more frequent symptoms.

^b^ Higher scores indicate more symptoms experienced.

^c^ Higher scores indicate more severe problems.

### Diagnostic Accuracy

#### Major Depressive Episode

The AUC for the PHQ-9 in the overall sample was 0.91 (95% CI, 0.88-0.94). The conventional cutoff point of at least 10 had the best balance of sensitivity and specificity (Table 3). The criteria of 5 or more symptoms rated at a score of least 2 favored specificity (0.94 [95% CI, 0.91-0.96] vs sensitivity of 0.67 [95% CI, 0.57-0.76])), while the criteria of 5 or more symptoms rated at a score of at least 1 favored sensitivity (0.94 [95% CI, 0.88-0.98] vs specificity of 0.64 [95% CI, 0.59-0.69]) (Table 3). For each cutoff, the positive likelihood ratios were greater than 2.00 and the negative likelihood ratios were less than 1.00. Additionally, the NPVs were 0.88 or greater, and the PPVs were 0.78 or less. Other cutoffs can be found in eTable 4 in Supplement 1.

**Table 3.** Diagnostic Accuracy for the Overall Sample

Measure by cutoff	No. with condition (%) (N = 499)	Accuracy (95% CI)					
		Sensitivity	Specificity	PPV	NPV	Positive LR	Negative LR
PHQ-9							
Total score ≥10^a^	88 (17.6)	0.86 (0.78-0.92)	0.83 (0.79-0.87)	0.57 (0.49-0.65)	0.96 (0.93-0.98)	5.11 (4.06-6.44)	0.17 (0.10-0.27)
≥5 Symptoms rated ≥2^a^^,^^b^	68 (13.6)	0.67 (0.57-0.76)	0.94 (0.91-0.96)	0.74 (0.64-0.83)	0.92 (0.89-0.94)	11.03 (7.31-16.64)	0.35 (0.27-0.47)
≥5 Symptoms rated ≥1^a^	96 (19.2)	0.94 (0.88-0.98)	0.64 (0.59-0.69)	0.40 (0.34-0.47)	0.98 (0.95-0.99)	2.63 (2.29-3.03)	0.09 (0.04-0.20)
GAD-7							
Total score ≥7	105 (21.0)	0.75 (0.67-0.82)	0.80 (0.75-0.84)	0.59 (0.52-0.67)	0.89 (0.85-0.92)	3.74 (2.98-4.70)	.31 (0.23-0.42)
Total score ≥10	77 (15.4)	0.55 (0.46-0.63)	0.89 (0.85-0.92)	0.66 (0.56-0.74)	0.84 (0.79-0.87)	4.94 (3.55-6.85)	.51 (0.42-0.61)
PC-PTSD-5							
Total score ≥3	32 (6.4)	0.67 (0.52-0.80)	0.87 (0.84-0.90)	0.36 (0.26-0.47)	0.96 (0.94-0.98)	5.27 (3.85-7.22)	0.38 (0.26-0.57)
Total score ≥4	27 (5.4)	0.56 (0.41-0.71)	0.92 (0.90-0.95)	0.44 (0.32-0.58)	0.95 (0.93-0.97)	7.46 (4.96-11.22)	0.47 (0.34-0.65)

Abbreviations: GAD-7, Generalized Anxiety Disorder–7; LR, likelihood ratio for positive or negative diagnostic test result; NPV, negative predictive value; PC-PTSD-5, Primary Care PTSD (Posttraumatic Stress Disorder) Screen for *DSM-5* (*Diagnostic and Statistical Manual of Mental Disorders* [Fifth Edition]); PHQ-9, Patient Health Questionnaire–9; PPV, positive predictive value.

^a^ Indicates at least 1 cardinal symptom endorsed.

^b^ Suicidal ideation is counted if present regardless of duration.

#### Anxiety Disorders

The AUC for the GAD-7 was 0.85 (95% CI, 0.81-0.88). The cutoff with the best balance between sensitivity and specificity (>0.70 for both) was at least 7 (eTable 10 in Supplement 1). The conventional cutoff score of at least 10 had a low sensitivity (0.55 [95% CI, 0.46-0.63]) and favored specificity (0.89 [95% CI, 0.85-0.92]) (Table 3). The positive likelihood ratios for each threshold were greater than 1.80 and the negative likelihood ratios were less than 0.70. Additionally, the NPVs were greater than 0.75, and the PPVs were less than 0.85.

#### Posttraumatic Stress Disorder

The AUC for the PC-PTSD-5 in the overall sample was 0.80 (95% CI, 0.72-0.87). A cutoff score of at least 3 had a sensitivity of 0.67 (95% CI, 0.52-0.80) with a specificity of 0.87 (95% CI, 0.84-0.90) (Table 3). A cutoff score of at least 4 yielded a poor sensitivity of 0.56 (95% CI, 0.41-0.71) with a specificity of 0.92 (95% CI, 0.90-0.95) (Table 3). The cutoff with the best balance between sensitivity and specificity, where both indices were at least 0.70, was a total score of at least 2 (eTable 23 in Supplement 1). The positive likelihood ratios for each threshold were greater than 2.00 and the negative likelihood ratios were less than 0.70. Additionally, the NPVs were greater than 0.90, and the PPVs were less than 0.60.

The GAD-7 had a higher AUC (0.85 [95% CI, 0.80-0.89]) than the PC-PTSD-5 (0.80 [95% CI, 0.72-0.87]; difference, 0.05 [*P* < .001]). The GAD-7 and PC-PTSD-5 combined modestly increased discriminability (AUC, 0.88 [95% CI, 0.80-0.96]). Using a likelihood ratio test, we found that the combined GAD-7 and PC-PTSD-5 model was a significantly better fit for the data than the GAD-7 (χ^2^_1_ = 26.61; *P* < .001) or the PC-PTSD-5 (χ^2^_1_ = 21.35; *P* < .001) alone, showing that the use of both questionnaires was better at identifying PTSD, as expected (eTable 31 in Supplement 1 for the multivariable regression model).

#### Diagnostic Accuracy in Patients With mTBI and PPCS Present or Absent

The AUCs were lower in the PPCS-present subgroup (≥0.75) compared with the PPCS-absent subgroup (≥0.76) and overall sample (≥0.80; difference, 0.01-0.13 percentage points) (Figure, A-C, and eTable 32 in Supplement 1 provide a comparison of AUC values for each questionnaire). Across cutoffs, sensitivity was higher (difference, 14-33 percentage points) and specificity was lower (difference, 5-65 percentage points) in the PPCS-present subgroup compared with the PPCS-absent subgroup and overall sample. However, PPV remained high for the PPCS-present subgroup, as it was offset by a higher base rate of mental health disorders (ie, 3 to 5 times higher). Also of note, the cutoff of at least 5 symptoms with a total score of at least 2 on the PHQ-9 was relatively robust to PPCS status, maintaining reasonable specificity (0.74 [95% CI, 0.62-0.84]). Relative to the PHQ-9 and GAD-7, the diagnostic accuracy of the PC-PTSD-PC was less affected by PPCS status (Figure, C vs A and B and Table 4).

**Figure.**
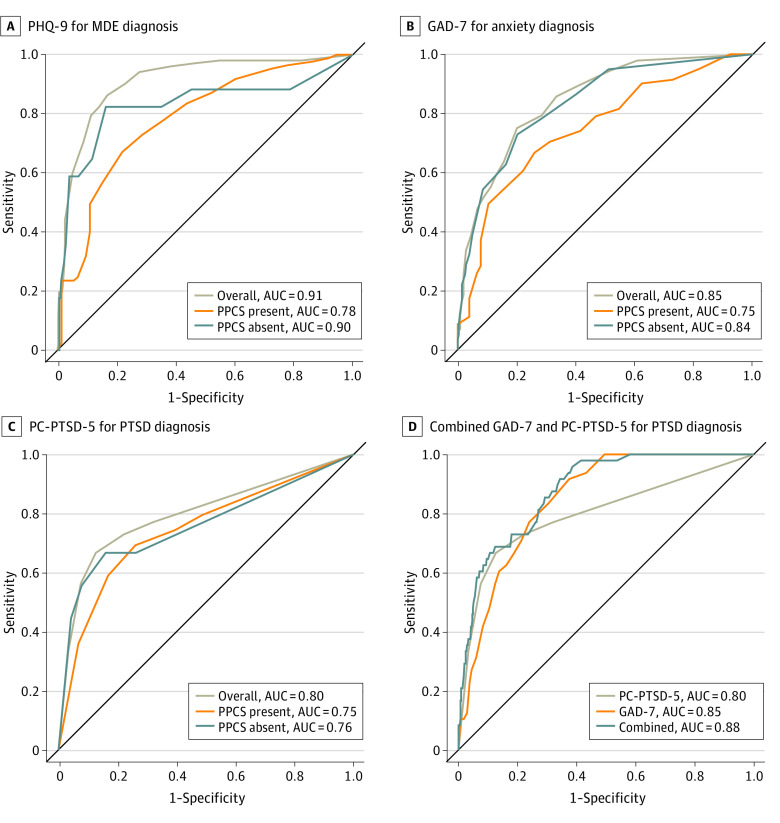
Receiver Operating Characteristics Curves for Each Screening Tool in the Overall Study Group and Subgroups With Persistent Postconcussive Symptoms (PPCS) Present and Absent Overall sample includes 499 participants; subgroup with PPCS present, 305 participants; and subgroup with PPCS absent, 194 participants. AUC indicates area under the curve; GAD-7, Generalized Anxiety Disorder–7; MDE, major depressive disorder; PC-PTSD-5, Primary Care PTSD (Posttramatic Stress Disorder) Screen for the *Diagnostic and Statistical Manual of Mental Disorders* (Fifth Edition); and PHQ-9, Patient Health Questionnaire–9.

**Table 4.** Diagnostic Accuracy for the Subgroups With PPCS Present and Absent

Measure by cutoff	No. with condition (%)	Accuracy (95% CI)					
		Sensitivity	Specificity	PPV	NPV	Positive LR	Negative LR
**Subgroup with PPCS present (n = 158)**							
PHQ-9							
Total score ≥10^a^	78 (49.4)	0.92 (0.84-0.97)	0.40 (0.28-0.52)	0.64 (0.55-0.72)	0.81 (0.64-0.92)	1.52 (1.25-1.85)	0.21 (0.10-0.44)
≥5 Symptoms rated ≥2^a^^,^^b^	61 (38.6)	0.72 (0.61-0.81)	0.74 (0.62-0.84)	0.76 (0.65-0.85)	0.69 (0.58-0.79)	2.76 (1.83-4.15)	0.38 (0.26-0.55)
≥5 Symptoms rated ≥1^a^	83 (52.5)	0.98 (0.92-1.00)	0.12 (0.06-0.22)	0.56 (0.48-0.65)	0.82 (0.48-0.98)	1.11 (1.02-1.22)	0.19 (0.04-0.86)
GAD-7							
Total score ≥7	73 (46.2)	0.90 (0.81-0.96)	0.38 (0.27-0.49)	0.60 (0.51-0.69)	0.78 (0.62-0.90)	1.45 (1.20-1.74)	0.26 (0.13-0.54)
Total score ≥10	60 (38.0)	0.74 (0.63-0.83)	0.58 (0.47-0.70)	0.65 (0.55-0.75)	0.68 (0.56-0.79)	1.78 (1.33-2.39)	0.44 (0.29-0.67)
PC-PTSD-5							
Total score ≥3	27 (17.1)	0.69 (0.52-0.83)	0.74 (0.65-0.82)	0.47 (0.33-0.60)	0.88 (0.80-0.94)	2.66 (1.84-3.84)	0.42 (0.26-0.67)
Total score ≥4	23 (14.6)	0.59 (0.42-0.74)	0.83 (0.75-0.89)	0.53 (0.38-0.69)	0.86 (0.78-0.92)	3.51 (2.18-5.66)	0.49 (0.34-0.72)
**Subgroup with PPCS absent (n = 341)**							
PHQ-9							
Total score ≥10^a^	10 (2.9)	0.59 (0.33-0.82)	0.93 (0.90-0.95)	0.30 (0.16-0.49)	0.98 (0.95-0.99)	8.29 (4.73-14.50)	0.44 (0.25-0.78)
≥5 Symptoms rated ≥2^a^^,^^b^	7 (2.1)	0.41 (0.18-0.67)	0.98 (0.96-0.99)	0.58 (0.28-0.85)	0.97 (0.94-0.99)	26.68 (9.44-75.40)	0.60 (0.40-0.89)
≥5 Symptoms rated ≥1^a^	13 (3.8)	0.76 (0.50-0.93)	0.76 (0.71-0.80)	0.14 (0.08-0.23)	0.98 (0.96-1.00)	3.18 (2.29-4.41)	0.31 (0.13-0.73)
GAD-7							
Total score ≥7	32 (9.4)	0.54 (0.41-0.67)	0.91 (0.88-0.94)	0.57 (0.43-0.70)	0.91 (0.87-0.94)	6.37 (4.07-9.98)	0.50 (0.38-0.66)
Total score ≥10	17 (5.0)	0.29 (0.18-0.42)	0.97 (0.94-0.99)	0.68 (0.46-0.85)	0.87 (0.82-0.90)	10.16 (4.60-22.43)	0.73 (0.62-0.86)
PC-PTSD-5							
Total score ≥3	5 (1.5)	0.56 (0.21-0.86)	0.92 (0.89-0.95)	0.16 (0.05-0.34)	0.99 (0.97-1.00)	7.09 (3.55-14.16)	0.48 (0.23-1.00)
Total score ≥4	4 (1.2)	0.44 (0.14-0.79)	0.96 (0.93-0.98)	0.22 (0.06-0.48)	0.98 (0.96-0.99)	10.54 (4.32-25.73)	0.58 (0.32-1.04)

Abbreviations: GAD-7, Generalized Anxiety Disorder–7; LR, likelihood ratio for positive or negative diagnostic test result; NPV, negative predictive value; PC-PTSD-5, Primary Care PTSD (Posttraumatic Stress Disorder) Screen for *DSM-5* (*Diagnostic and Statistical Manual of Mental Disorders* [Fifth Edition]); PHQ-9, Patient Health Questionnaire–9; PPCS, persistent postconcussive symptoms; PPV, positive predictive value.

^a^ Indicates at least 1 cardinal symptom endorsed.

^b^ Suicidal ideation is counted if present regardless of duration.

## Discussion

The present study supports the diagnostic accuracy of the PHQ-9 for detecting MDE, GAD-7 for detecting anxiety disorders, and PC-PTSD-5 for detecting PTSD in patients with mTBI. The overall diagnostic accuracy was high (AUC ≥0.80) for all questionnaires.^45^ Similar to previous studies in primary care patients and TBIs of all severities, the PHQ-9 had outstanding accuracy (≥0.90).^17,29,45^ The AUCs for the GAD-7 and PC-PTSD-5 were somewhat lower (0.80-0.85) compared with previous literature.^21,22,23,33,42^ Our study strengthens the evidence for the PHQ-9 and GAD-7 in a large and more representative sample with mTBI than previous studies and supports the diagnostic accuracy of the PC-PTSD-5.

When investigating previously recommended cutoffs for each questionnaire, we found some deviations in sensitivity and specificity. For the PHQ-9, we obtained results similar to those reported in previous studies at most cutoffs, including a total score of at least 10.^17,28,29^ However, when considering 5 or more symptoms rated with a score of at least 1 (with ≥1 symptom being a cardinal symptom), specificity was lower compared with that found by Fann et al.^29^ Further, the recommended cutoffs of 7 and 10 for the GAD-7 and 3 and 4 for the PC-PTSD-5 yielded lower sensitivity and higher specificity values compared with prior studies.^19,20,21,27,32^

To our knowledge, this is the first study to evaluate the diagnostic accuracy of the PHQ-9, GAD-7, and PC-PTSD-5 in adults with vs without PPCS. The AUC values were lower but still acceptable (≥0.75) in participants with PPCS present. Despite reduced specificity in the PPCS-present subgroup, PPV was similar in the PPCS-present subgroup and the full sample because the base rate of mental health disorders was much higher in this group (relative risk, 2.96-10.97). In other words, a positive mental health screen result was associated with comparable likelihood of mental health diagnosis in the PPCS-present subgroup and full sample, across cutoffs. This finding suggests that the same cutoffs could be used for all patients with mTBI. The co-occurrence of PPCS and mental health disorders is not surprising because of their symptom overlap, but also because PPCS can be distressing and, in turn, emotional distress can exacerbate PPCS.^12,54^

Although the PHQ-9 total score cutoff of at least 10 and the algorithm of 5 or more symptoms with a score of at least 2 performed similarly in the full sample, the latter was more robust in the PPCS-present subgroup, providing a reason to favor it in specialty concussion clinic settings. The relatively simple and familiar cutoff of at least 10 could be used in primary care with only a modest loss of PPV. Further, because PPCS and mental health symptoms overlap, a detailed clinical assessment is needed to disentangle which symptoms are attributable to a mental health diagnosis, such as by querying the onset and course to determine whether they align better with that diagnosis.

Our analyses also revealed that the GAD-7 was slightly better at detecting the presence of PTSD compared with the PC-PTSD-5. The AUC for the GAD-7 was greater than 0.80, similar to a previous study of patients seen in primary care settings.^33^ Combining both questionnaires (PC-PTSD-5 and GAD-7) compared with each questionnaire alone was associated with a modest increase in the ability to detect PTSD (difference in AUC, 0.05; *P* < .001), but was likely not clinically meaningful. Using only the GAD-7 to screen for both anxiety disorders and PTSD is most efficient. However, if a high specificity (>0.85) is important, using the PC-PTSD-5 with a higher cutoff—or combining both questionnaires—may be more appropriate.

### Limitations

This study has some limitations. Although past or current mental health problems were not inclusion criteria, a self-selection bias may have resulted in an overrepresentation of patients with these characteristics. However, our sample is likely representative of patients who seek follow-up care for mTBI and undergo mental health screening for clinical purposes. Older adults and Black and Hispanic patients were underrepresented in our sample. Our sample size was larger than those of previous diagnostic accuracy studies in mTBI, but still relatively small for establishing the optimal cutoffs and precise confidence intervals of the estimates.^55^ Because of the low prevalence of PTSD (48 cases [9.6%]) and of all mental health disorders in the PPCS-absent subgroup, the estimates of diagnostic accuracy in these analyses had wider 95% CIs. Another limitation is that psychodiagnostic interviewing to determine the “true state” of mental health disorders was performed by research personnel rather than physicians or psychologists. Standardized assessor training, use of a highly structured and validated diagnostic interview (the MINI), and ongoing supervision by a psychologist mitigate this concern. The MINI is a relatively streamlined diagnostic interview and may overidentify cases of depression compared with the Structured Clinical Interview for *DSM-5*.^56^

## Conclusions

The findings of this diagnostic study suggest that the PHQ-9 can be used to accurately identify MDE, the GAD-7 can be used to identify anxiety disorders, and the PC-PTSD-5 can be used to identify PTSD after mTBI, regardless of PPCS burden. Concurrent PPCS lower their specificity, but given the higher prevalence of mental health disorders in this patient group, a positive mental health screen result should similarly trigger a formal diagnostic evaluation. The combination of GAD-7 and PC-PTSD-PC optimize detection of PTSD after mTBI but are marginally better than the GAD-7 alone. Future research should corroborate optimal test cutoffs for this population.

## Notes

Supplement 1. **eMethods.** Case Ascertainment **eFigure.** STARD Flowchart of Participants **eTable 1.** Recommended Cutoff Scores for the Screening Tools **eTable 2.** Severity of Depression According to PHQ-9 **eTable 3.** PHQ-9 Diagnostic Accuracy for Overall Sample (N = 499) **eTable 4.** True and False Positive and Negative Rates for Each Cutoff for PHQ-9 in Overall Sample (N = 499) **eTable 5.** PHQ-9 Diagnostic Accuracy for Sample With PPCS Present (n = 158) **eTable 6.** True and False Positive and Negative Rates for Each Cutoff for PHQ-9 for Sample With PPCS Present (n = 158) **eTable 7.** PHQ-9 Diagnostic Accuracy for Sample With PPCS Absent (n = 341) **eTable 8.** True and False Positive and Negative Rates for Each Cutoff for PHQ-9 for Sample With PPCS Absent (n = 341) **eTable 9.** Severity of Anxiety According to GAD-7 **eTable 10.** Diagnostic Accuracy of GAD-7 to Diagnose at Least 1 Anxiety Disorder for Overall Sample (N = 499) **eTable 11.** True and False Positive and Negative Rates for Each Cutoff for GAD-7 to Diagnose at Least 1 Anxiety Disorder for Overall Sample (N = 499) **eTable 12.** Diagnostic Accuracy of GAD-7 to Diagnose at Least 1 Anxiety Disorder for Sample With PPCS Present (n = 158) **eTable 13.** True and False Positive and Negative Rates for Each Cutoff for GAD-7 to Diagnose at Least 1 Anxiety Disorder for Sample With PPCS Present (n = 158) **eTable 14.** Diagnostic Accuracy of GAD-7 to Diagnose at Least 1 Anxiety Disorder for Sample With PPCS Absent (n = 341) **eTable 15.** True and False Positive and Negative Rates for Each Cutoff for GAD-7 to Diagnose at Least 1 Anxiety Disorder for Sample With PPCS Absent (n = 341) **eTable 16.** Diagnostic Accuracy of GAD-7 to Diagnose Generalized Anxiety Disorder for Overall Sample (N = 499) **eTable 17.** True and False Positive and Negative Rates for Each Cutoff for GAD-7 to Diagnose at Least 1 Anxiety Disorder for Overall Sample (N = 499) **eTable 18.** Diagnostic Accuracy of GAD-7 to Diagnose Generalized Anxiety Disorder for Sample With PPCS Present (n = 158) **eTable 19.** True and False Positive and Negative Rates for Each Cutoff for GAD-7 to Diagnose Generalized Anxiety Disorder for Sample With PPCS Present (n = 158) **eTable 20.** Diagnostic Accuracy of GAD-7 to Diagnose Generalized Anxiety Disorder for Sample With PPCS Absent (n = 341) **eTable 21.** True and False Positive and Negative Rates for Each Cutoff for GAD-7 to Diagnose Generalized Anxiety Disorder for Sample With PPCS Absent (n = 341) **eTable 22.** Severity of PTSD According to PC-PTSD-5 **eTable 23.** Diagnostic Accuracy of PC-PTSD-5 for Overall Sample (N = 499) **eTable 24.** True and False Positive and Negative Rates for Each Cutoff for PC-PTSD-5 for Overall Sample (N = 499) **eTable 25.** Diagnostic Accuracy of PC-PTSD-5 for Sample With PPCS Present (n = 158) **eTable 26.** True and False Positive and Negative Rates for Each Cutoff for PC-PTSD-5 for Sample With PPCS Present (n = 158) **eTable 27.** Diagnostic Accuracy of PC-PTSD-5 for Sample With PPCS Absent (n = 341) **eTable 28.** True and False Positive and Negative Rates for Each Cutoff for Sample With PPCS Absent (n = 341) **eTable 29.** Diagnostic Accuracy of GAD-7 to Diagnose PTSD for Overall Sample (N = 499) **eTable 30.** True and False Positive and Negative Rates for Each Cutoff for GAD-7 to Diagnose PTSD for Overall Sample (N = 499) **eTable 31.** Multivariable Regression Model **eTable 32.** AUC Comparison for Each Screening Questionnaire in the Overall, PPCS Present, and PPCS Absent Samples
eReferences

Supplement 2.
Data Sharing Statement
