# Supplementary material for: Diagnostic Accuracy of Mental Health Screening Tools After Mild Traumatic Brain Injury
Source: JAMA Netw Open. 2024 Jul 23;7(7):e2424076. doi: 10.1001/jamanetworkopen.2024.24076 (PMC11267412; doi:10.1001/jamanetworkopen.2024.24076)
Supplement: Supplement 1. — eMethods. Case Ascertainment eFigure. STARD Flowchart of Participants eTable 1. Recommended Cutoff Scores for the Screening Tools eTable 2. Severity of Depression According to PHQ-9 eTable 3. PHQ-9 Diagnostic Accuracy for Overall Sample (N = 499) eTable 4. True and False Positive and Negative Rates for Each Cutoff for PHQ-9 in Overall Sample (N = 499) eTable 5. PHQ-9 Diagnostic Accuracy for Sample With PPCS Present (n = 158) eTable 6. True and False Positive and Negative Rates for Each Cutoff for PHQ-9 for Sample With PPCS Present (n = 158) eTable 7. PHQ-9 Diagnostic Accuracy for Sample With PPCS Absent (n = 341) eTable 8. True and False Positive and Negative Rates for Each Cutoff for PHQ-9 for Sample With PPCS Absent (n = 341) eTable 9. Severity of Anxiety According to GAD-7 eTable 10. Diagnostic Accuracy of GAD-7 to Diagnose at Least 1 Anxiety Disorder for Overall Sample (N = 499) eTable 11. True and False Positive and Negative Rates for Each Cutoff for GAD-7 to Diagnose at Least 1 Anxiety Disorder for Overall Sample (N = 499) eTable 12. Diagnostic Accuracy of GAD-7 to Diagnose at Least 1 Anxiety Disorder for Sample With PPCS Present (n = 158) eTable 13. True and False Positive and Negative Rates for Each Cutoff for GAD-7 to Diagnose at Least 1 Anxiety Disorder for Sample With PPCS Present (n = 158) eTable 14. Diagnostic Accuracy of GAD-7 to Diagnose at Least 1 Anxiety Disorder for Sample With PPCS Absent (n = 341) eTable 15. True and False Positive and Negative Rates for Each Cutoff for GAD-7 to Diagnose at Least 1 Anxiety Disorder for Sample With PPCS Absent (n = 341) eTable 16. Diagnostic Accuracy of GAD-7 to Diagnose Generalized Anxiety Disorder for Overall Sample (N = 499) eTable 17. True and False Positive and Negative Rates for Each Cutoff for GAD-7 to Diagnose at Least 1 Anxiety Disorder for Overall Sample (N = 499) eTable 18. Diagnostic Accuracy of GAD-7 to Diagnose Generalized Anxiety Disorder for Sample With PPCS Present (n = 158) eTable 19. True and Fa [file jamanetwopen-e2424076-s001.pdf]

Supplementary Online Content

Gitaari M, Mikolić A, Panenka WJ, Silverberg ND. Diagnostic accuracy of mental health screening tools after mild traumatic brain injury. *JAMA Netw Open*. 2024;7(7):e2424076. doi:10.1001/jamanetworkopen.2024.24076

eMethods. Case Ascertainment
eFigure. STARD Flowchart of Participants
eTable 1. Recommended Cutoff Scores for the Screening Tools
eTable 2. Severity of Depression According to PHQ-9
eTable 3. PHQ-9 Diagnostic Accuracy for Overall Sample (N = 499)
eTable 4. True and False Positive and Negative Rates for Each Cutoff for PHQ-9 in Overall Sample (N = 499)
eTable 5. PHQ-9 Diagnostic Accuracy for Sample With PPCS Present (n = 158)
eTable 6. True and False Positive and Negative Rates for Each Cutoff for PHQ-9 for Sample With PPCS Present (n = 158)
eTable 7. PHQ-9 Diagnostic Accuracy for Sample With PPCS Absent (n = 341)
eTable 8. True and False Positive and Negative Rates for Each Cutoff for PHQ-9 for Sample With PPCS Absent (n = 341)
eTable 9. Severity of Anxiety According to GAD-7
eTable 10. Diagnostic Accuracy of GAD-7 to Diagnose at Least 1 Anxiety Disorder for Overall Sample (N = 499)
eTable 11. True and False Positive and Negative Rates for Each Cutoff for GAD-7 to Diagnose at Least 1 Anxiety Disorder for Overall Sample (N = 499)
eTable 12. Diagnostic Accuracy of GAD-7 to Diagnose at Least 1 Anxiety Disorder for Sample With PPCS Present (n = 158)
eTable 13. True and False Positive and Negative Rates for Each Cutoff for GAD-7 to Diagnose at Least 1 Anxiety Disorder for Sample With PPCS Present (n = 158)
eTable 14. Diagnostic Accuracy of GAD-7 to Diagnose at Least 1 Anxiety Disorder for Sample With PPCS Absent (n = 341)
eTable 15. True and False Positive and Negative Rates for Each Cutoff for GAD-7 to Diagnose at Least 1 Anxiety Disorder for Sample With PPCS Absent (n = 341)
eTable 16. Diagnostic Accuracy of GAD-7 to Diagnose Generalized Anxiety Disorder for Overall Sample (N = 499)
eTable 17. True and False Positive and Negative Rates for Each Cutoff for GAD-7 to Diagnose at Least 1 Anxiety Disorder for Overall Sample (N = 499)
eTable 18. Diagnostic Accuracy of GAD-7 to Diagnose Generalized Anxiety Disorder for Sample With PPCS Present (n = 158)
eTable 19. True and False Positive and Negative Rates for Each Cutoff for GAD-7 to Diagnose Generalized Anxiety Disorder for Sample With PPCS Present (n = 158)
eTable 20. Diagnostic Accuracy of GAD-7 to Diagnose Generalized Anxiety Disorder for Sample With PPCS Absent (n = 341)
eTable 21. True and False Positive and Negative Rates for Each Cutoff for GAD-7 to Diagnose Generalized Anxiety Disorder for Sample With PPCS Absent (n = 341)
eTable 22. Severity of PTSD According to PC-PTSD-5
eTable 23. Diagnostic Accuracy of PC-PTSD-5 for Overall Sample (N = 499)
eTable 24. True and False Positive and Negative Rates for Each Cutoff for PC-PTSD-5 for Overall Sample (N = 499)
eTable 25. Diagnostic Accuracy of PC-PTSD-5 for Sample With PPCS Present (n = 158)
eTable 26. True and False Positive and Negative Rates for Each Cutoff for PC-PTSD-5 for Sample With PPCS Present (n = 158)
eTable 27. Diagnostic Accuracy of PC-PTSD-5 for Sample With PPCS Absent (n = 341)

© 2024 Gitaari M et al. *JAMA Network Open*.

eTable 28. True and False Positive and Negative Rates for Each Cutoff for Sample With PPCS Absent (n = 341)
eTable 29. Diagnostic Accuracy of GAD-7 to Diagnose PTSD for Overall Sample (N = 499)
eTable 30. True and False Positive and Negative Rates for Each Cutoff for GAD-7 to Diagnose PTSD for Overall Sample (N = 499)
eTable 31. Multivariable Regression Model
Table 32. AUC Comparison for Each Screening Questionnaire in the Overall, PPCS Present, and PPCS Absent Samples
eReferences

This supplementary material has been provided by the authors to give readers additional information about their work.

© 2024 Gitaari M et al. *JAMA Network Open*.

eMethods. Case Ascertainment

Case ascertainment was completed by research assistants screening patient medical charts using a method developed by Pozzato et al.1,2 which is derived from the WHO Neurotrauma Task Force definition of mTBI. According to this definition, an mTBI diagnosis requires 1 or more of the following: loss of consciousness for ≤ 30 minutes, posttraumatic amnesia < 24 hours, confusion, disorientation, or other transient neurological problems (i.e., nonsurgical intracranial lesions, seizure, or focal signs). Additionally, a Glasgow Coma Scale (GCS) score of 13-15 is required thirty minutes after injury or upon arrival at a health care facility. Using an algorithm based on these guidelines research assistants identified potentially eligible participants with probable or possible mTBI based on emergency department chart review, as described below.

Probable mTBI: A GCS score of 13 or 14 upon arrival at a hospital with a feasible mechanism of head trauma caused by an external force, or with a GCS score of 13 or 14 at hospital arrival, or other signs and symptoms described in the WHO Neurotrauma Task Force Guidelines. If present, alcohol or drug intoxication does not account for the signs of mTBI

Possible mTBI: Lack of clinical indicators of probable mTBI with a feasible mechanism of head trauma caused by an external force. However, an emergency physician diagnosed the patient with an mTBI or reported the presence of 2 or more post-concussion symptoms and clinical suspicion of mTBI (head CT ordered or queried but unclear loss of consciousness or post-traumatic amnesia). If present, alcohol or drug intoxication does not account for the signs of mTBI

All prospective participants with probable or possible mTBI then underwent a structured interview based on the WHO Neurotrauma Task Force definition of mTBI to confirm their diagnosis. The interview was conducted by trained research assistants who queried about loss of consciousness, confusion or disorientation, and post-traumatic amnesia, as well as possible confounding factors for these findings.

© 2024 Gitaari M et al. *JAMA Network Open*.

eFigure. STARD Flowchart of Participants

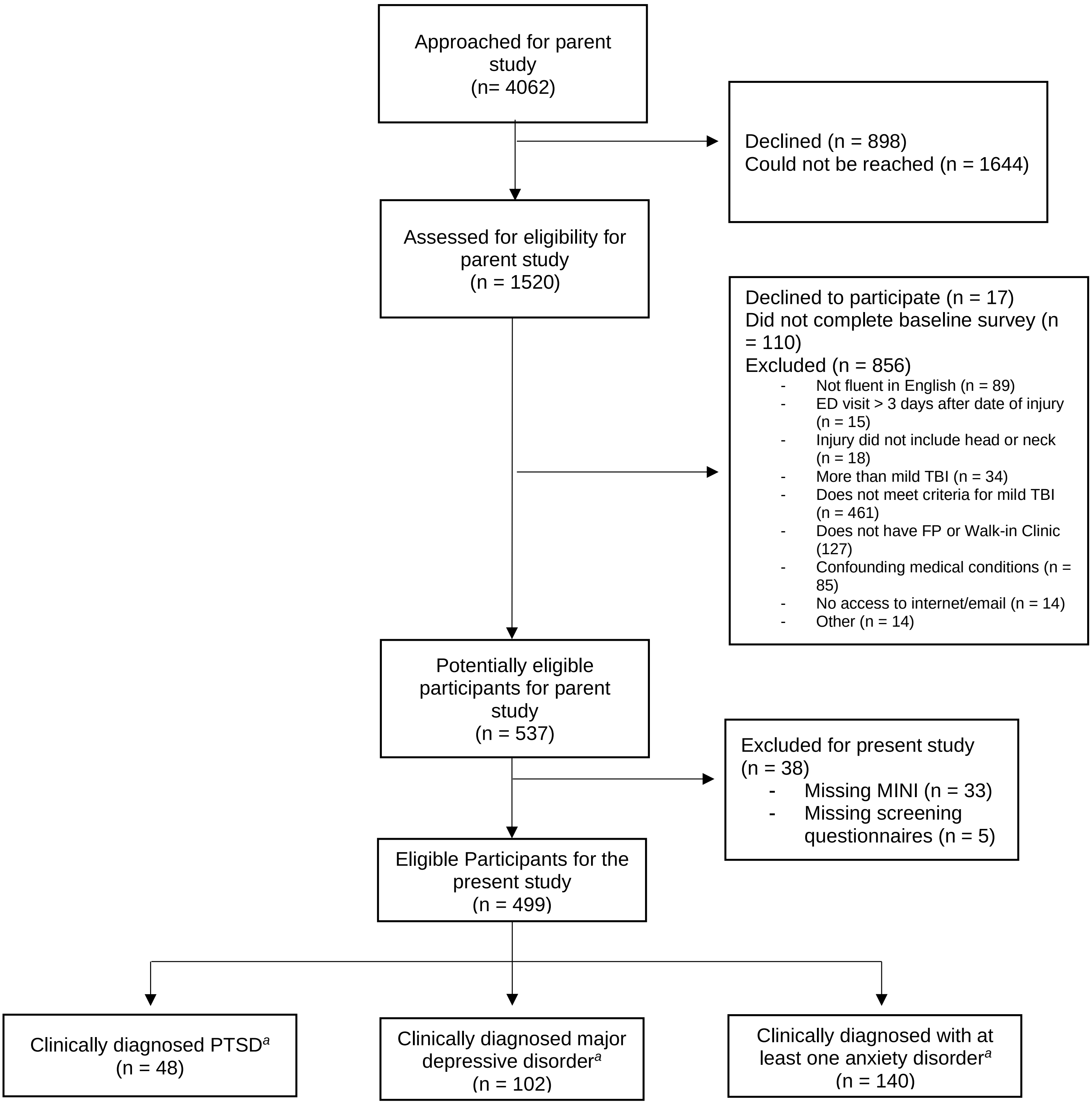

Abbreviations: ED, emergency department; FP, family physician; TBI, traumatic brain injury; MINI, Mini International Neuropsychiatric Interview; PTSD, posttraumatic stress disorder

[a]Participants could have had more than one mental health disorder.

© 2024 Gitaari M et al. *JAMA Network Open*.

eTable 1. Recommended Cutoff Scores for the Screening Tools

Source	Measure	Recommended Cut-off	Sensitivity	Specificity
[3–5]	*PHQ-9*	≥ 10	.88-.92	.60-.90
[4]		≥ 10[a]	.73	.95
[3,4]		5 or more symptoms rated ≥ 2. [a, b]	.60	.98
[4]		5 or more symptoms rated ≥ 1 [a, b]	.93	.89
[6]	*GAD-7*	≥ 7	.91	.68
[7]		≥ 10	.62-.89	.82-.87
[8,9]	*PC-PTSD-5*	≥ 3	.90-.95	.80-.85
[10]		≥ 4	1.00	.85

Abbreviations: PHQ-9, Patient Health Questionnaire-9; GAD-7, Generalised Anxiety Disorder-7; PC-PTSD-5, Primary Care-PTSD-5
[a] With at least one cardinal symptom endorsed.
[b] Suicidal ideation is counted no matter its duration if present.

eTable 2. Severity of Depression According to PHQ-9

	Overall (N = 499)	PPCS + (*n* = 158)	PPCS – (*n* = 341)
**Severity**	**No. (%)**	**No. (%)**	**No. (%)**
Minimal [a]	217 (43.5)	4 (2.5)	213 (62.5)
Mild [b]	127(25.5)	32 (20.3)	95 (27.9)
Moderate [c]	74 (14.8)	49 (31.0)	25 (7.3)
Moderately Severe [d]	52 (10.4)	47 (29.7)	5 (1.5)
Severe [e]	29 (5.8)	26 (16.5)	3 (0.9)

Abbreviations: PPCS +, persistent postconcussive symptoms present; PPCS -, persistent postconcussive symptoms absent
[a] *Score of 0-4*
[b] *Score of 5-9*
[c] *Score of 10-14*
[d] *Score of 15-19*
[e] *Score of 20-27*

eTable 3. PHQ-9 Diagnostic Accuracy for Overall Sample (N = 499)

Cut-Off	No. with Depression (%)	Sensitivity (95%CI)	Specificity (95%CI)	PPV (95%CI)	NPV (95%CI)	LR + (95%CI)	LR- (95%CI)
≥ 5	99 (19.8)	.97 (0.92-0.99)	.54 (0.49-0.59)	.35 (0.30-0.41)	.99 (0.96-1.00)	2.11 (1.88-2.35)	.05 (0.02-0.17)
≥ 6	98 (19.6)	.96 (0.90-0.99)	.61 (0.56-0.66)	.39 (0.33-0.45)	.98 (0.96-1.00)	2.49 (2.19-2.84)	.06 (0.02-0.17)
≥ 7	97 (19.4)	.95 (0.89-0.98)	.67 (0.62-0.72)	.43 (0.36-0.49)	.98 (0.96-0.99)	2.88 (2.49-3.34)	.07 (0.03-0.17)
≥ 8	96 (19.2)	.94 (0.88-0.98)	.72 (0.68-0.77)	.47 (0.40-0.54)	.98 (0.96-0.99)	3.40 (2.88-4.01)	.08 (0.02-0.18)
≥ 9	92 (18.4)	.90 (0.83-0.95)	.77 (0.73-0.81)	.50 (0.43-0.58)	.97 (0.94-0.98)	3.93 (3.25-4.76)	.13 (0.07-0.23)
≥ 10	88 (17.6)	.86 (0.78-0.92)	.83 (0.79-0.87)	.57 (0.49-0.65)	.96 (0.93-0.98)	5.11 (4.06-6.44)	.17 (0.10-0.27)
≥ 10[a]	88 (17.6)	.86 (0.78-0.92)	.83 (0.79-0.87)	.57 (0.49-0.65)	.96 (0.93-0.98)	5.11 (4.06-6.44)	.17 (0.10-0.27)
≥ 11	84 (16.8)	.82 (0.74-0.89)	.86 (0.82-0.89)	.60 (0.51-0.68)	.95 (0.92-0.97)	5.84 (3.85-6.04)	.21 (0.13-0.31)
≥ 12	81 (16.2)	.79 (0.70-0.87)	.89 (0.85-0.92)	.64 (0.55-0.73)	.94 (0.92-0.96)	7.01 (5.23-9.38)	.23 (0.11-0.31)
≥ 13	72 (14.4)	.71 (0.61-0.79)	.91 (0.88-0.94)	.67 (0.58-0.76)	.92 (0.89-0.95)	8.01 (5.70-11.25)	.32 (0.24-0.44)
≥ 14	67 (13.4)	.66 (0.56-0.75)	.93 (0.90-0.95)	.71 (0.60-0.79)	.91 (0.88-0.94)	9.31 (6.35-13.67)	.37 (0.28-0.48)
≥ 15	61 (12.2)	.60 (0.50-0.69)	.95 (0.92-0.97)	.75 (0.64-0.84)	.90 (0.87-0.93)	11.87 (7.53-18.72)	.42 (0.33-0.54)
≥ 16	51 (10.2)	.50 (0.40-0.60)	.96 (0.94-0.98)	.78 (0.67-0.88)	.88 (0.85-0.91)	14.18 (8.18-24.57)	.52 (0.43-0.63)
5 or more symptoms rated ≥ 2. [a,b]	68 (13.6)	.67 (0.57-0.76)	.94 (0.91-0.96)	.74 (0.64-0.83)	.92 (0.89-0.94)	11.03 (7.31-16.64)	0.35 (0.27-0.47)
5 or more symptoms rated ≥1.[a]	96 (19.2)	.94 (0.88-0.98)	.64 (0.59-0.69)	.40 (0.34-0.47)	.98 (0.95-0.99)	2.63 (2.29-3.03)	0.09 (0.04-0.20)

Abbreviations: PPV, Positive Predictive Value; NPV, Negative Predictive Value, LR +, likelihood ratio of a positive test; LR -, likelihood ratio of a negative test; No., number; CI, confidence interval

[a] With at least one cardinal symptom endorsed

[b] Suicidal ideation is counted no matter its duration if present

© 2024 Gitaari M et al. *JAMA Network Open*.

eTable 4. True and False Positive and Negative Rates for Each Cutoff for PHQ-9 in Overall Sample (N = 499)

Cut-Off	TP	FP	TN	FN
≥ *5*	99	183	214	3
≥ *6*	98	153	244	4
≥ *7*	97	131	266	5
≥ *8*	96	110	287	6
≥ *9*	92	91	306	10
≥ *10*	88	67	330	14
≥ *11*	84	56	341	18
≥ *12*	81	45	352	21
≥ *13*	72	35	362	30
≥ *14*	67	28	369	35
≥ *15*	61	20	377	41
≥ *16*	51	14	383	51
≥ *10*[a]	88	67	330	14
5 or more symptoms rated ≥ *2.* [a,b]	68	24	373	34
5 or more symptoms rated ≥*1.*[a]	96	142	255	6

Abbreviations: TP, true positive; FP, false positive; TN, true negative; FN, false negative
[a] With at least one cardinal symptom endorse
[b] Suicidal ideation is counted no matter its duration if present

© 2024 Gitaari M et al. *JAMA Network Open*.

eTable 5. PHQ-9 Diagnostic Accuracy for Sample With PPCS Present (n = 158)

Cut-Off	No. with Depression (%)	Sensitivity (95%CI)	Specificity (95%CI)	PPV (95%CI)	NPV (95%CI)	LR + (95%CI)	LR- (95%CI)
≥ 5	85 (53.8)	1.00	.05 (0.02-0.13)	.55 (0.47-0.63)	1.00	1.06 (1.00-1.12)	.00
≥ 6	84 (53.2)	.99 (0.94-1.00)	.08 (0.03-0.17)	.56 (0.47-0.64)	.86 (0.42-1.00)	1.08 (1.00-1.16)	.14 (0.02-1.16)
≥ 7	83 (52.5)	.98 (0.92-1.00)	.14 (0.07-0.24)	.57 (0.48-0.65)	.83 (0.52-0.98)	1.13 (1.03-1.25)	.17 (0.04-0.76)
≥ 8	82 (51.9)	.96 (0.90 -0.99)	.22 (0.13-0.33)	.59 (0.50-0.67)	.84 (0.60-0.97)	1.24 (1.09-1.40)	.16 (0.05-0.53)
≥ 9	81 (51.3)	.95 (0.88-0.99)	.27 (0.18-0.39)	.60 (0.52-0.69)	.83 (0.63-0.95)	1.31 (1.13-1.52)	.17 (0.06-0.48)
≥ 10	78 (49.4)	.92 (0.84-0.97)	.40 (0.28-0.52)	.64 (0.55-0.72)	.81 (0.64-0.92)	1.52 (1.25-1.85)	.21 (0.10-0.44)
≥ 10[a]	78 (49.4)	.92 (0.84-0.97)	.40 (0.28-0.52)	.64 (0.55-0.72)	.81 (0.64-0.92)	1.52 (1.25-1.85)	.21 (0.10-0.44)
≥ 11	74 (46.8)	.87 (0.78-0.93)	.48 (0.36-0.60)	.66 (0.57-0.75)	.76 (0.61-0.87)	1.67 (1.32-2.12)	.27 (0.15-0.49)
≥ 12	71 (44.9)	.84 (0.74-0.91)	.56 (0.44-0.68)	.69 (0.59-0.78)	.75 (0.61-0.85)	1.91 (1.45-2.51)	.29 (0.17-0.49)
≥ 13	66 (41.8)	.78 (0.67-0.86)	.64 (0.52-0.75)	.72 (0.61-0.81)	.71 (0.59-0.82)	2.18 (1.57-3.03)	.35 (0.23-0.53)
≥ 14	62 (39.2)	.73 (0.62-0.82)	.71 (0.59-0.81)	.75 (0.64-0.84)	.69 (0.58-0.79)	2.54 (1.73-3.72)	.38 (0.26-0.55)
≥ 15	57 (36.1)	.67 (0.56-0.77)	.78 (0.67-0.87)	.78 (0.67-0.87)	.67 (0.56-0.77)	3.06 (1.94-4.84)	.42 (0.30-0.58)
≥ 16	48 (30.4)	.56 (0.45-0.67)	.85 (0.75-0.92)	.81 (0.69-0.90)	.63 (0.52-0.72)	3.75 (2.11-6.66)	.51 (0.39-0.67)
5 or more symptoms rated ≥ 2. [a,b]	61 (38.6)	.72 (0.61-0.81)	.74 (0.62-0.84)	.76 (0.65-0.85)	.69 (0.58-0.79)	2.76 (1.83-4.15)	0.38 (0.26-0.55)
5 or more symptoms rated ≥1.[a]	83 (52.5)	.98 (0.92-1.00)	.12 (0.06-0.22)	.56 (0.48-0.65)	.82 (0.48-0.98)	1.11 (1.02-1.22)	0.19 (0.04-0.86)

Abbreviations: PPV, Positive Predictive Value; NPV, Negative Predictive Value, LR +, likelihood ratio of a positive test; LR -, likelihood ratio of a negative test; No., number; CI, confidence interval
[a] With at least one cardinal symptom endorsed
[b] Suicidal ideation is counted no matter its duration if present

© 2024 Gitaari M et al. *JAMA Network Open*.

eTable 6. True and False Positive and Negative Rates for Each Cutoff for PHQ-9 for Sample With PPCS Present (n = 158)

Cut-Off	TP	FP	TN	FN
≥ *5*	85	69	4	0
≥ *6*	84	67	6	1
≥ *7*	83	63	10	2
≥ *8*	82	57	16	3
≥ *9*	81	53	20	4
≥ *10*	78	44	29	7
≥ *11*	74	38	35	11
≥ *12*	71	32	41	14
≥ *13*	66	26	47	19
≥ *14*	62	21	52	23
≥ *15*	57	16	57	28
≥ *16*	48	11	62	37
≥ *10*[a]	78	44	29	7
5 or more symptoms rated ≥ 2. [a,b]	61	19	54	24
5 or more symptoms rated ≥1.[a]	83	64	9	2

Abbreviations: TP, true positive; FP, false positive; TN, true negative; FN, false negative
[a] With at least one cardinal symptom endorsed
[b] Suicidal ideation is counted no matter its duration if present

© 2024 Gitaari M et al. *JAMA Network Open*.

eTable 7. PHQ-9 Diagnostic Accuracy for Sample With PPCS Absent (n = 341)

Cut-Off	No. with Depression (%)	Sensitivity (95%CI)	Specificity (95%CI)	PPV (95%CI)	NPV (95%CI)	LR + (95%CI)	LR- (95%CI)
≥ *5*	14 (4.1)	.82 (0.57-0.96)	.65 (0.59-0.70)	.11 (0.06-0.18)	.99 (0.96-1.00)	2.34 (1.80-3.05)	.27 (0.10-0.76)
≥ *6*	14 (4.1)	.82 (0.57-0.96)	.73 (0.68-0.78)	.14 (0.08-0.22)	.99 (0.96-1.00)	3.10 (2.33-4.13)	.24 (0.09-0.67)
≥ *7*	14 (4.1)	.82 (0.57-0.96)	.79 (0.74-0.83)	.17 (0.10-0.27)	.99 (0.97-1.00)	3.92 (2.89-5.32)	0.22 (0.08-0.62)
≥ *8*	14 (4.1)	.82 (0.57-0.96)	.84 (0.79-0.88)	.21 (0.12-0.33)	.99 (0.97-1.00)	5.03 (3.62-7.00)	0.21 (0.08-0.59)
≥ *9*	11 (3.2)	.65 (0.38-0.86)	.88 (0.84-0.92)	.22 (0.12-0.37)	.98 (0.96-0.99)	5.52 (3.48-8.75)	0.40 (0.21-0.76)
≥ *10*	10 (2.9)	.59 (0.33-0.82)	.93 (0.90-0.95)	.30 (0.16-0.49)	.98 (0.95-0.99)	8.29 (4.73-14.50)	0.44 (0.25-0.78)
≥ *10*[a]	10 (2.9)	.59 (0.33-0.82)	.93 (0.90-0.95)	.30 (0.16-0.49)	.98 (0.95-0.99)	8.29 (4.73-14.50)	0.44 (0.25-0.78)
≥ *11*	10 (2.9)	.59 (0.33-0.82)	.94 (0.91-0.97)	.36 (0.19-0.56)	.98 (0.95-0.99)	10.59 (5.81-19.29)	.44 (0.25-0.77)
≥ *12*	10 (2.9)	.59 (0.33-0.82)	.96 (0.93-0.98)	.43 (0.23-0.66)	.98 (0.96-0.99)	14.66 (7.54-28.50)	.43 (0.24-0.76)
≥ *13*	6 (1.8)	.35 (0.14-0.62)	.97 (0.95-0.99)	.40 (0.16-0.68)	.97 (0.94-0.98)	12.71 (5.11-31.59)	.67 (0.47-0.95)
≥ *14*	5 (1.5)	.29 (0.10-0.56)	.98 (0.96-0.99)	.42 (0.15-0.72)	.96 (0.94-0.98)	13.61 (4.82-38.47)	.72 (0.53-0.98)
≥ *15*	4 (1.2)	.24 (0.07-0.50)	.99 (0.97-1.00)	.50 (0.16-0.84)	.96 (0.93-0.98)	19.06 (5.21-69.74)	.77 (0.59-1.01)
≥ *16*	3 (0.9)	.18 (0.04-0.43)	.99 (0.97-1.00)	.50 (0.12-0.88)	.96 (0.93-0.98)	19.06 (4.15-87.51)	.83 (0.67-1.04)
5 or more symptoms rated ≥ 2. [a,b]	7 (2.1)	.41 (0.18-0.67)	.98 (0.96-0.99)	.58 (0.28-0.85)	.97 (0.94-0.99)	26.68 (9.44-75.40)	0.60 (0.40-0.89)
5 or more symptoms rated ≥1.[a]	13 (3.8)	.76 (0.50-0.93)	.76 (0.71-0.80)	.14 (0.08-0.23)	.98 (0.96-1.00)	3.18 (2.29-4.41)	0.31 (0.13-0.73)

Abbreviations: PPV, Positive Predictive Value; NPV, Negative Predictive Value, LR +, likelihood ratio of a positive test; LR -, likelihood ratio of a negative test; No., number; CI, confidence interval

[a] With at least one cardinal symptom endorsed

[b] Suicidal ideation is counted no matter its duration if present

© 2024 Gitaari M et al. *JAMA Network Open*.

eTable 8. True and False Positive and Negative Rates for Each Cutoff for PHQ-9 for Sample With PPCS Absent (n = 341)

Cut-Off	TP	FP	TN	FN
≥ *5*	14	114	210	3
≥ *6*	14	86	238	3
≥ *7*	14	68	256	3
≥ *8*	14	53	271	3
≥ *9*	11	38	286	6
≥ *10*	10	23	301	7
≥ *11*	10	18	306	7
≥ *12*	10	13	311	7
≥ *13*	6	9	315	11
≥ *14*	5	7	317	12
≥ *15*	4	4	320	13
≥ *16*	3	3	321	14
≥ *10*[a]	10	23	301	7
5 or more symptoms rated ≥ *2.* [a,b]	7	5	319	10
5 or more symptoms rated ≥*1.*[a]	13	78	246	4

Abbreviations: TP, true positive; FP, false positive; TN, true negative; FN, false negative
[a] With at least one cardinal symptom endorsed
[b] Suicidal ideation is counted no matter its duration if present

eTable 9. Severity of Anxiety According to GAD-7

	**Overall (N = 499)**	**PPCS + (*n* = 158)**	**PPCS – (*n* = 341)**
**Severity**	**No. (%)**	**No. (%)**	**No. (%)**
Minimal [a]	259 (51.9)	18 (11.4)	241 (70.7)
Mild [b]	123(24.6)	48 (30.4)	75 (22.0)
Moderate [c]	60 (12.0)	44 (27.8)	16 (4.7)
Severe [d]	57 (11.4)	48 (30.4)	9 (2.6)

Abbreviations: PPCS +, persistent postconcussive symptoms present; PPCS -, persistent postconcussive symptoms absent
[a] *Score of 0-4*
[b] *Score of 5-9*
[c] *Score of 10-14*
[d] *Score of 15-21*

© 2024 Gitaari M et al. *JAMA Network Open*.

eTable 10. Diagnostic Accuracy of GAD-7 to Diagnose at Least 1 Anxiety Disorder for Overall Sample (N = 499)

Cut-Off	No. with Anxiety (%)	Sensitivity (95%CI)	Specificity (95%CI)	PPV (95%CI)	NPV (95%CI)	LR + (95%CI)	LR- (95%CI)
≥ *3*	132 (26.5)	.94 (0.89-0.98)	.49 (0.43-0.54)	.42 (0.36-0.47)	.96 (0.92-0.98)	1.84 (1.65-2.05)	0.12 (0.06-0.23)
≥ *4*	125 (25.1)	.89 (0.83-0.94)	.59 (0.54-0.64)	.46 (0.40-0.52)	.93 (0.89-0.96)	2.20 (1.91-2.52)	0.18 (0.11-0.29)
≥ *5*	120 (24.0)	.86 (0.79-0.91)	.67 (0.61-0.71)	.50 (0.44-0.56)	.92 (0.88-0.95)	2.56 (2.18-3.01)	.21 (0.14-0.32)
≥ *6*	111 (22.2)	.79 (0.72-0.86)	.72 (0.67-0.76)	.52 (0.45-0.59)	.90 (0.86-0.93)	2.79 (2.32-3.36)	.29 (0.21-0.40)
≥ *7*	105 (21.0)	.75 (0.67-0.82)	.80 (0.75-0.84)	.59 (0.52-67)	.89 (0.85-0.92)	3.74 (2.98-4.70)	.31 (0.23-0.42)
≥ *8*	89 (17.8)	.64 (0.55-0.72)	.84 (0.80-0.88)	.61 (0.53-0.69)	.86 (0.81-0.89)	4.08 (3.11-5.35)	.43 (0.35-0.54)
≥ *9*	83 (16.6)	.59 (0.51-0.68)	.87 (0.83-0.90)	.64 (0.55-0.72)	.85 (0.80-0.88)	4.53 (3.36-6.11)	.47 (0.38-0.57)
≥ *10*	77 (15.4)	.55 (0.46-0.63)	.89 (0.85-0.92)	.66 (0.56-0.74)	.84 (0.79-0.87)	4.94 (3.55-6.85)	.51 (0.42-0.61)
≥ *11*	72 (14.4)	.51 (0.43-0.60)	.91 (0.88-0.94)	.70 (0.60-0.79)	.83 (0.79-0.86)	5.96 (4.10-8.65)	0.53 (0.45-0.63)
≥ *12*	67 (13.4)	.48 (0.39-0.56)	.93 (0.90-0.96)	.74 (0.63-0.82)	.82 (0.78-0.86)	7.16 (4.69-10.93)	.56 (0.48-0.66)
≥ *13*	62 (12.4)	.44 (0.36-0.53)	.94 (0.91-0.96)	.75 (0.64-0.84)	.81 (0.77-0.85)	7.57 (4.80-11.93)	.59 (0.51-0.69)
≥ *14*	54 (10.8)	.39 (0.30-0.47)	.96 (0.93-0.97)	.77 (0.66-0.86)	.80 (0.76-0.84)	8.65 (5.13-14.59)	.64 (0.56-0.73)
≥ *15*	47 (9.4)	.34 (0.26-0.42)	.97 (0.95-0.99)	.82 (0.70-0.91)	.79 (0.75-0.83)	12.05 (6.27-23.18)	.68 (0.61-0.77)

Abbreviations: PPV, Positive Predictive Value; NPV, Negative Predictive Value, LR +, likelihood ratio of a positive test; LR -, likelihood ratio of a negative test; No., number; CI, confidence interval

© 2024 Gitaari M et al. *JAMA Network Open*.

eTable 11. True and False Positive and Negative Rates for Each Cutoff for GAD-7 to Diagnose at Least 1 Anxiety Disorder for Overall Sample (N = 499)

**Cut-Off**	**TP**	**FP**	**TN**	**FN**
≥ 3	132	184	175	8
≥ 4	125	146	213	15
≥ *5*	120	120	239	20
≥ *6*	111	102	257	29
≥ *7*	105	72	287	35
≥ *8*	89	56	303	51
≥ *9*	83	47	312	57
≥ *10*	77	40	319	63
≥ 11	72	31	328	68
≥ 12	67	24	335	73
≥ 13	62	21	338	78
≥ 14	54	16	343	86
≥ 15	47	10	349	93

Abbreviations: TP, true positive; FP, false positive; TN, true negative; FN, false negative

© 2024 Gitaari M et al. *JAMA Network Open*.

eTable 12. Diagnostic Accuracy of GAD-7 to Diagnose at Least 1 Anxiety Disorder for Sample With PPCS Present (n = 158)

Cut-Off	No. with Anxiety (%)	Sensitivity (95%CI)	Specificity (95%CI)	PPV (95%CI)	NPV (95%CI)	LR + (95%CI)	LR- (95%CI)
≥ *3*	81 (51.3)	1.00	.08 (0.03-0.16)	.53 (0.45-0.61)	1.00	1.08 (1.02-1.16)	0.00
≥ *4*	79 (50.0)	.98 (0.91-1.00)	.13 (0.06-0.23)	.54 (0.46-0.62)	.83 (0.52-0.98)	1.12 (1.02-1.23)	0.19 (0.04-0.84)
≥ *5*	77 (48.7)	.95 (0.88-0.99)	.18 (0.10-0.29)	.55 (0.46-0.63)	.78 (0.52-0.94)	1.16 (1.03-1.31)	.27 (0.09-0.79)
≥ *6*	74 (46.8)	.91 (0.83-0.96)	.27 (0.18-0.39)	.57 (0.48-0.66)	.75 (0.55-0.89)	1.26 (1.08-1.46)	.32 (0.14-0.70)
≥ *7*	73 (46.2)	.90 (0.81-0.96)	.38 (0.27-0.49)	.60 (0.51-0.69)	.78 (0.62-0.90)	1.45 (1.20-1.74)	.26 (0.13-0.54)
≥ *8*	66 (41.8)	.81 (0.71-0.89)	.45 (0.34-0.57)	.61 (0.51-0.70)	.70 (0.55-0.82)	1.49 (1.19-1.88)	.41 (0.24-0.68)
≥ *9*	64 (40.5)	.79 (0.69-0.87)	.53 (0.42-0.65)	.64 (0.54-0.73)	.71 (0.57-0.82)	1.69 (1.30-2.20)	.39 (0.25-0.63)
≥ *10*	60 (38.0)	.74 (0.63-0.83)	.58 (0.47-0.70)	.65 (0.55-0.75)	.68 (0.56-0.79)	1.78 (1.33-2.39)	.44 (0.29-0.67)
≥ 11	57 (36.1)	.70 (0.59-0.80)	.69 (0.57-0.79)	.70 (0.59-0.80)	.68 (0.57-0.79)	2.26 (1.57-3.24)	.43 (0.30-0.62)
≥ 12	54 (34.2)	.67 (0.55-0.77)	.74 (0.63-0.83)	.73 (0.61-0.83)	.68 (0.57-0.78)	2.57 (1.17-3.86)	0.45 (0.32-0.63)
≥ 13	49 (31.0)	.60 (0.49-0.71)	.78 (0.67-0.87)	.74 (0.62-0.84)	.65 (0.55-0.75)	2.74 (1.74-4.32)	0.51 (0.38-0.68)
≥ 14	44 (27.8)	.54 (0.43-0.65)	.84 (0.74-0.92)	.79 (0.66-0.88)	.64 (0.54-0.73)	3.49 (2.00-6.08)	0.54 (0.42-0.70)
≥ 15	40 (25.3)	.49 (0.38-0.61)	.90 (0.81-0.95)	.83 (0.70-0.93)	.63 (0.53-0.72)	4.75 (2.38-9.50)	0.56 (0.45-0.71)

Abbreviations: PPV, Positive Predictive Value; NPV, Negative Predictive Value, LR +, likelihood ratio of a positive test; LR -, likelihood ratio of a negative test; No., number; CI, confidence interval

eTable 13. True and False Positive and Negative Rates for Each Cutoff for GAD-7 to Diagnose at Least 1 Anxiety Disorder for Sample With PPCS Present (n = 158)

Cut-Off	TP	FP	TN	FN
≥ 3	81	71	6	0
≥ 4	79	67	10	2
≥ *5*	77	63	14	4
≥ *6*	74	56	21	7
≥ *7*	73	48	29	8
≥ *8*	66	42	35	15
≥ *9*	64	36	41	17
≥ *10*	60	32	45	21
≥ 11	57	24	53	24
≥ 12	54	20	57	27
≥ 13	49	17	60	32
≥ 14	44	12	65	37
≥ 15	40	8	69	41

Abbreviations: TP, true positive; FP, false positive; TN, true negative; FN, false negative

© 2024 Gitaari M et al. *JAMA Network Open*.

eTable 14. Diagnostic Accuracy of GAD-7 to Diagnose at Least 1 Anxiety Disorder for Sample With PPCS Absent (n = 341)

Cut-Off	No. with Anxiety (%)	Sensitivity (95%CI)	Specificity (95%CI)	PPV (95%CI)	NPV (95%CI)	LR + (95%CI)	LR- (95%CI)
≥ *3*	51 (15.0)	.86 (0.75-0.94)	.60 (0.54-0.66)	.31 (0.24-0.39)	.95 (0.91-0.98)	2.16 (1.81-2.57)	.23 (0.12-0.43)
≥ *4*	46 (13.5)	.78 (0.65-0.88)	.72 (0.66-0.77)	.37 (0.28-0.46)	.94 (0.90-0.97)	2.78 (2.21-3.51)	.31 (0.19-0.50)
≥ *5*	43 (12.6)	.73 (0.60-0.84)	.80 (0.75-0.84)	.43 (0.33-0.53)	.93 (0.89-0.96)	3.61 (2.73-4.77)	.34 (0.22-0.52)
≥ *6*	37 (10.9)	.63 (0.49-0.75)	.84 (0.79-0.88)	.45 (0.34-0.56)	.91 (0.87-0.95)	3.84 (2.77-5.35)	.45 (0.32-0.62)
≥ *7*	32 (9.4)	.54 (0.41-0.67)	.91 (0.88-0.94)	.57 (0.43-0.70)	.91 (0.87-0.94)	6.37 (4.07-9.98)	.50 (0.38-0.66)
≥ *8*	23 (6.7)	.39 (0.27-0.53)	.95 (0.92-0.97)	.62 (0.45-0.78)	.88 (0.84-0.92)	7.85 (4.30-14.34)	.64 (0.52-0.79)
≥ *9*	19 (5.6)	.32 (0.21-0.46)	.96 (0.93-0.98)	.63 (0.44-0.80)	.87 (0.83-0.91)	8.26 (4.15-16.42)	.71 (0.59-0.84)
≥ *10*	17 (5.0)	.29 (0.18-0.42)	.97 (0.94-0.99)	.68 (0.46-0.85)	.87 (0.82-0.90)	10.16 (4.60-22.43)	.73 (0.62-0.86)
≥ *11*	15 (4.4)	.25 (0.15-0.38)	.98 (0.95-0.99)	.68 (0.45-0.86)	.86 (0.82-0.90)	10.24 (4.37-24.01)	.76 (0.66-0.89)
≥ *12*	13 (3.8)	.22 (0.12-0.35)	.99 (0.96-1.00)	.76 (0.50-0.93)	.86 (0.82-0.89)	15.53 (5.25-45.97)	.79 (0.69-0.91)
≥ *13*	13 (3.8)	.22 (0.12-0.35)	.99 (0.96-1.00)	.76 (0.50-0.93)	.86 (0.82-0.89)	15.53 (5.25-45.97)	.79 (0.69-0.91)
≥ *14*	10 (2.9)	.17 (0.08-0.29)	.99 (0.96-1.00)	.71 (0.42-0.92)	.85 (0.81-0.89)	11.95 (3.88-36.81)	.84 (0.75-0.95)
≥ *15*	7 (2.1)	.12 (0.05-0.23)	.99 (0.97-1.00)	.78 (0.40-0.97)	.84 (0.80-0.88)	16.73 (3.56-78.52)	.89 (0.81-0.98)

Abbreviations: PPV, Positive Predictive Value; NPV, Negative Predictive Value, LR +, likelihood ratio of a positive test; LR -, likelihood ratio of a negative test; No., number; CI, confidence interval

eTable 15. True and False Positive and Negative Rates for Each Cutoff for GAD-7 to Diagnose at Least 1 Anxiety Disorder for Sample With PPCS Absent (n = 341)

Cut-Off	TP	FP	TN	FN
≥ 3	51	113	169	8
≥ 4	46	79	203	13
≥ *5*	43	57	225	16
≥ *6*	37	46	236	22
≥ *7*	32	24	258	27
≥ *8*	23	14	268	36
≥ *9*	19	11	271	40
≥ *10*	17	8	274	42
≥ 11	15	7	275	44
≥ 12	13	4	278	46
≥ 13	13	4	278	46
≥ 14	10	4	278	49
≥ 15	7	2	280	52

Abbreviations: TP, true positive; FP, false positive; TN, true negative; FN, false negative

© 2024 Gitaari M et al. *JAMA Network Open*.

eTable 16. Diagnostic Accuracy of GAD-7 to Diagnose Generalized Anxiety Disorder for Overall Sample (N = 499)

Cut-Off	No. with Anxiety (%)	Sensitivity (95%CI)	Specificity (95%CI)	PPV (95%CI)	NPV (95%CI)	LR + (95%CI)	LR- (95%CI)
≥ *3*	69 (13.8)	.99 (0.92-1.00)	.42 (0.38-0.47)	.22 (0.17-0.27)	.99 (0.97-1.00)	1.71 (1.57-1.87)	.03 (0.00-0.24)
≥ *4*	68 (13.6)	.97 (0.90-1.00)	.53 (0.48-0.57)	.25 (0.20-0.31)	.99 (0.97-1.00)	2.05 (1.84-2.29)	.05 (0.01-0.21)
≥ *5*	65 (13.0)	.93 (0.84-0.8)	.59 (0.54-0.64)	.27 (0.22-0.33)	.98 (0.96-0.99)	2.28 (2.00-2.60)	.12 (0.05-0.28)
≥ *6*	62 (12.4)	.89 (0.79-0.95)	.65 (0.60-0.69)	.29 (0.23-0.36)	.97 (0.95-0.99)	2.52 (2.16-2.93)	.18 (0.09-0.34)
≥ *7*	59 (11.8)	.84 (0.74-0.92)	.72 (0.68-0.77)	.33 (0.26-0.41)	.97 (0.94-0.98)	3.06 (2.55-3.68)	.22 (0.13-0.37)
≥ *8*	53 (10.6)	.76 (0.64-0.85)	.79 (0.74-0.82)	.37 (0.29-0.45)	.95 (0.92-0.97)	3.53 (2.82-4.42)	.31 (0.20-0.47)
≥ *9*	50 (10.0)	.71 (0.59-0.82)	.81 (0.77-0.85)	.38 (0.30-0.47)	.95 (0.92-0.97)	3.83 (2.99-4.90)	.35 (0.24-0.51)
≥ *10*	46 (9.2)	.66 (0.53-0.77)	.83 (0.80-0.87)	.39 (0.30-0.49)	.94 (0.91-0.96)	3.97 (3.03-5.21)	.41 (0.30-0.57)
≥ *11*	42 (8.4)	.60 (0.48-0.72)	.86 (0.82-0.89)	.41 (0.31-0.51)	.93 (0.90-0.95)	4.22 (3.12-5.70)	.47 (0.35-0.62)
≥ *12*	38 (7.6)	.54 (0.42-0.66)	.88 (0.84-0.91)	.42 (0.32-0.53)	.92 (0.89-0.95)	4.39 (3.15-6.12)	.52 (0.40-0.67)
≥ *13*	35 (7.0)	.50 (0.38-0.62)	.89 (0.85-0.92)	.42 (0.31-0.54)	.92 (0.88-0.94)	4.47 (3.13-6.37)	.56 (0.44-0.71)
≥ *14*	31 (6.2)	.44 (0.32-0.57)	.91 (0.88-0.93)	.44 (0.32-0.57)	.91 (0.88-0.93)	4.87 (3.27-7.25)	.61 (0.50-0.76)
≥ *15*	27 (5.4)	.39 (0.27-0.51)	.93 (0.90-0.95)	.47 (0.34-0.61)	.90 (0.87-0.93)	5.52 (3.50-8.69)	.66 (0.55-0.80)

Abbreviations: PPV, Positive Predictive Value; NPV, Negative Predictive Value, LR +, likelihood ratio of a positive test; LR -, likelihood ratio of a negative test; No., number; CI, confidence interval

© 2024 Gitaari M et al. *JAMA Network Open*.

eTable 17. True and False Positive and Negative Rates for Each Cutoff for GAD-7 to Diagnose at Least 1 Anxiety Disorder for Overall Sample (N = 499)

Cut-Off	TP	FP	TN	FN
≥ 3	69	247	182	1
≥ 4	68	203	226	2
≥ *5*	65	175	254	5
≥ *6*	62	151	278	8
≥ *7*	59	118	311	11
≥ *8*	53	92	337	17
≥ *9*	50	80	349	20
≥ *10*	46	71	358	24
≥ 11	42	61	368	28
≥ 12	38	53	376	32
≥ 13	35	48	381	35
≥ 14	31	39	390	39
≥ 15	27	30	399	43

Abbreviations: TP, true positive; FP, false positive; TN, true negative; FN, false negative

© 2024 Gitaari M et al. *JAMA Network Open*.

eTable 18. Diagnostic Accuracy of GAD-7 to Diagnose Generalized Anxiety Disorder for Sample With PPCS Present (n = 158)

**Cut-Off**	**No. with Anxiety (%)**	**Sensitivity (95%CI)**	**Specificity (95%CI)**	**PPV (95%CI)**	**NPV (95%CI)**	**LR + (95%CI)**	**LR- (95%CI)**
≥ *3*	47 (29.7)	1.00	.05 (0.02-0.11)	.31 (0.24-0.39)	1.00	1.06 (1.01-1.11)	.00
≥ *4*	46 (29.1)	.98 (0.89-1.00)	.10 (0.05-0.17)	.32 (0.24-0.40)	.92 (0.62-1.00)	1.09 (1.01-1.17)	0.21 (0.03-1.62)
≥ *5*	45 (28.5)	.96 (0.85-0.99)	.14 (0.08-0.22)	.32 (0.25-0.41)	.89 (0.65-0.99)	1.12 (1.02-1.23)	.30 (0.07-1.23)
≥ *6*	44 (27.8)	.94 (0.82-0.99)	.23 (0.15-0.31)	.34 (0.26-0.49)	.89 (0.72-0.98)	1.21 (1.07-1.37)	.28 (0.09-0.89)
≥ *7*	44 (27.8)	.94 (0.82-0.99)	.31 (0.22-0.40)	.36 (0.28-0.46)	.92 (0.78-0.98)	1.35 (1.17-1.56)	.21 (0.07-0.65)
≥ *8*	41 (25.9)	.87 (0.74-0.95)	.40 (0.30-0.49)	.38 (0.29-0.48)	.88 (0.76-0.95)	1.45 (1.20-1.74)	.32 (0.15-0.70)
≥ *9*	40 (25.3)	.85 (0.72-0.94)	.46 (0.36-0.56)	.40 (0.30-0.50)	.88 (0.77-0.95)	1.57 (1.28-1.94)	.32 (0.16-0.66)
≥ *10*	37 (23.4)	.79 (0.64-0.89)	.50 (0.41-0.60)	.40 (0.30-0.51)	.85 (0.74-0.92)	1.59 (1.25-2.02)	.42 (0.24-0.75)
≥ *11*	34 (21.5)	.72 (0.57-0.84)	.58 (0.48-0.67)	.42 (0.31-0.53)	.83 (0.73-0.91)	1.71 (1.29-2.26)	.48 (0.29-0.78)
≥ *12*	32 (20.3)	.68 (0.53-0.81)	.62 (0.52-0.71)	.43 (0.32-0.55)	.82 (0.72-0.90)	1.80 (1.32-2.45)	.51 (0.33-0.80)
≥ *13*	29 (18.4)	.62 (0.46-0.75)	.67 (0.57-0.75)	.44 (0.32-0.57)	.80 (0.71-0.88)	1.85 (1.31-2.62)	.57 (0.39-0.85)
≥ *14*	26 (16.5)	.55 (0.40-0.70)	.73 (0.64-0.81)	.46 (0.33-0.60)	.79 (0.70-0.87)	2.05 (1.37-3.05)	.61 (0.44-0.86)
≥ *15*	22 (13.9)	.47 (0.32-0.62)	.77 (0.68-0.84)	.46 (0.31-0.61)	.77 (0.68-0.85)	2.00 (1.27-3.15)	.69 (0.52-0.93)

Abbreviations: PPV, Positive Predictive Value; NPV, Negative Predictive Value, LR +, likelihood ratio of a positive test; LR -, likelihood ratio of a negative test; No., number; CI, confidence interval

eTable 19. True and False Positive and Negative Rates for Each Cutoff for GAD-7 to Diagnose Generalized Anxiety Disorder for Sample With PPCS Present (n = 158)

**Cut-Off**	**TP**	**FP**	**TN**	**FN**
≥ 3	47	105	6	0
≥ 4	46	100	11	1
≥ *5*	45	95	16	2
≥ *6*	44	86	25	3
≥ *7*	44	77	34	3
≥ *8*	41	67	44	6
≥ *9*	40	60	51	7
≥ *10*	37	55	56	10
≥ 11	34	47	64	13
≥ 12	32	42	69	15
≥ 13	29	37	74	18
≥ 14	26	30	81	21
≥ 15	22	26	85	25

Abbreviations: TP, true positive; FP, false positive; TN, true negative; FN, false negative

© 2024 Gitaari M et al. *JAMA Network Open*.

eTable 20. Diagnostic Accuracy of GAD-7 to Diagnose Generalized Anxiety Disorder for Sample With PPCS Absent (n = 341)

**Cut-Off**	**No. with Anxiety (%)**	**Sensitivity (95%CI)**	**Specificity (95%CI)**	**PPV (95%CI)**	**NPV (95%CI)**	**LR + (95%CI)**	**LR- (95%CI)**
≥ *3*	22 (6.5)	.96 (0.78-1.00)	.55 (0.50-0.61)	.13 (0.08-0.19)	.99 (0.97-1.00)	2.14 (1.84-2.49)	0.08 (0.01-0.54)
≥ *4*	22 (6.5)	.96 (0.78-1.00)	.68 (0.62-0.73)	.18 (0.11-0.25)	1.00 (0.97-1.00)	2.95 (2.46.-3.54)	0.06 (0.01-0.44)
≥ *5*	20 (5.9)	.87 (0.66-0.97)	.75 (0.70-0.80)	.20 (0.13-0.29)	.99 (0.96-1.00)	3.46 (2.70-4.42)	.17 (0.06-0.50)
≥ *6*	18 (5.3)	.78 (0.56-0.93)	.80 (0.75-0.84)	.22 (0.13-0.32)	.98 (0.96-0.99)	3.83 (2.82-5.20)	.27 (0.13-0.59)
≥ *7*	15 (4.4)	.65 (0.43-0.84)	.87 (0.83-0.91)	.27 (0.16-0.40)	.97 (0.95-0.99)	5.06 (3.35-7.65)	.40 (0.23-0.70)
≥ *8*	12 (3.5)	.52 (0.31-0.73)	.92 (0.89-0.95)	.32 (0.18-0.50)	.96 (0.94-0.98)	6.64 (3.86-11.42)	.52 (0.34-0.80)
≥ *9*	10 (2.9)	.43 (0.23-0.66)	.94 (0.90-0.96)	.33 (0.17-0.53)	.96 (0.93-0.98)	6.91 (3.68-12.98)	.60 (0.42-0.86)
≥ *10*	9 (2.6)	.39 (0.20-0.61)	.95 (0.92-0.97)	.36 (0.18-0.57)	.96 (0.93-0.98)	7.78 (3.87-15.64)	.64 (0.46-0.89)
≥ *11*	8 (2.3)	.35 (0.16-0.57)	.96 (0.93-0.98)	.36 (0.17-0.95)	.95 (0.92-0.97)	7.90 (3.70-16.87)	.68 (0.51-0.92)
≥ *12*	6 (1.8)	.26 (0.10-0.48)	.97 (0.94-0.98)	.35 (0.14-0.62)	.95 (0.92-0.97)	7.54 (3.07-18.55)	.77 (0.60-0.98)
≥ *13*	6 (1.8)	.26 (0.10-0.48)	.97 (0.94-0.98)	.35 (0.14-0.62)	.95 (0.92-0.97)	7.54 (3.07-18.55)	.77 (0.60-0.98)
≥ *14*	5 (1.5)	.22 (0.07-0.44)	.97 (0.95-0.99)	.36 (0.13-0.65)	.94 (0.91-0.97)	7.68 (2.80-21.05)	.81 (0.65-1.00)
≥ *15*	5 (1.5)	.22 (0.07-0.44)	.99 (0.97-1.00)	.56 (0.21-0.86)	.95 (0.92-0.97)	17.28 (4.98-60.01)	.79 (0.64-0.98)

Abbreviations: PPV, Positive Predictive Value; NPV, Negative Predictive Value, LR +, likelihood ratio of a positive test; LR -, likelihood ratio of a negative test; No., number; CI, confidence interval

© 2024 Gitaari M et al. *JAMA Network Open*.

eTable 21. True and False Positive and Negative Rates for Each Cutoff for GAD-7 to Diagnose Generalized Anxiety Disorder for Sample With PPCS Absent (n = 341)

Cut-Off	TP	FP	TN	FN
≥ 3	22	142	176	1
≥ 4	22	103	215	1
≥ *5*	20	80	238	3
≥ *6*	18	65	253	5
≥ *7*	15	41	277	8
≥ *8*	12	25	293	11
≥ *9*	10	20	298	13
≥ *10*	9	16	302	14
≥ 11	8	14	304	15
≥ 12	6	11	307	17
≥ 13	6	11	307	17
≥ 14	5	9	309	18
≥ 15	5	4	314	18

Abbreviations: TP, true positive; FP, false positive; TN, true negative; FN, false negative

eTable 22. Severity of PTSD According to PC-PTSD-5

	Overall (N = 499)	PPCS + (*n* = 158)	PPCS– (*n* = 341)
**Severity**	**No. (%)**	**No. (%)**	**No. (%)**
0	317 (63.5)	69 (43.7)	248 (72.7)
1	47 (9.4)	13 (8.2)	34 (10.0)
2	46 (9.2)	18 (11.4)	28 (8.2)
3	28 (5.6)	16 (10.1)	13 (3.8)
4	30 (6.0)	21 (13.3)	9 (2.6)
5	31 (6.2)	22 (13.9)	9 (2.6)

Abbreviations: PPCS +, persistent postconcussive symptoms present; PPCS -, persistent postconcussive symptoms absent; No., number

© 2024 Gitaari M et al. *JAMA Network Open*.

eTable 23. Diagnostic Accuracy of PC-PTSD-5 for Overall Sample (N = 499)

Cut-Off	No. with PTSD (%)	Sensitivity (95%CI)	Specificity (95%CI)	PPV (95%CI)	NPV (95%CI)	LR + (95%CI)	LR- (95%CI)
≥ *1*	37 (7.41)	.77 (0.63-0.88)	.68 (0.63-0.72)	.20 (0.15-0.27)	.97 (0.94-0.98)	2.40 (1.95-2.94)	.34 (0.20-0.57)
≥ *2*	35 (7.0)	.73 (0.58-0.85)	.78 (0.74-0.82)	.26 (0.19-0.34)	.96 (0.94-0.98)	3.29 (2.58-4.20)	.35 (0.22-0.56)
≥ *3*	32 (6.4)	.67 (0.52-0.80)	.87 (0.84-0.90)	.36 (0.26-0.47)	.96 (0.94-0.98)	5.27 (3.85-7.22)	.38 (0.26-0.57)
≥ *4*	27 (5.4)	.56 (0.41-0.71)	.92 (0.90-0.95)	.44 (0.32-0.58)	.95 (0.93-0.97)	7.46 (4.96-11.22)	.47 (0.34-0.65)
≥ *5*	16 (3.2)	.33 (0.20-0.48)	.97 (0.95-0.98)	.52 (0.33-0.70)	.93 (0.90-0.95)	10.02 (5.29-18.98)	.69 (0.56-0.84)

Abbreviations: PTSD, posttraumatic stress disorder; PPV, Positive Predictive Value; NPV, Negative Predictive Value; LR +, likelihood ratio of a positive test; LR -, likelihood ratio of a negative test; No., number; CI, confidence interval

eTable 24. True and False Positive and Negative Rates for Each Cutoff for PC-PTSD-5 for Overall Sample (N = 499)

Cut-Off	TP	FP	TN	FN
≥ *1*	37	145	306	11
≥ *2*	35	100	351	13
≥ *3*	32	57	394	16
≥ *4*	27	34	417	21
≥ *5*	16	15	436	32

Abbreviations: PPV, Positive Predictive Value; NPV, Negative Predictive Value

eTable 25. Diagnostic Accuracy of PC-PTSD-5 for the Sample With PPCS Present (n = 158)

Cut-Off	No. with PTSD (%)	Sensitivity (95%CI)	Specificity (95%CI)	PPV (95%CI)	NPV (95%CI)	LR + (95%CI)	LR- (95%CI)
≥ *1*	31 (19.6)	.79 (0.64-0.91)	.51 (0.42-0.61)	.35 (0.25-0.46)	.88 (0.78-0.95)	1.63 (1.28-2.08)	.40 (0.21-0.76)
≥ *2*	29 (18.4)	.74 (0.58-0.87)	.61 (0.51-0.69)	.38 (0.27-0.50)	.88 (0.79-0.94)	1.88 (1.41-2.51)	.42 (0.24-0.74)
≥ *3*	27 (17.1)	.69 (0.52-0.83)	.74 (0.65-0.82)	.47 (0.33-0.60)	.88 (0.80-0.94)	2.66 (1.84-3.84)	.42 (0.26-0.67)
≥ *4*	23 (14.6)	.59 (0.42-0.74)	.83 (0.75-0.89)	.53 (0.38-0.69)	.86 (0.78-0.92)	3.51 (2.18-5.66)	.49 (0.34-0.72)
≥ *5*	14 (8.9)	.36 (0.21-0.53)	.93 (0.87-0.97)	.64 (0.41-0.83)	.82 (0.74-0.88)	5.34 (2.42.11.76)	.69 (0.54-0.87)

Abbreviations: PTSD, posttraumatic stress disorder; PPV, Positive Predictive Value; NPV, Negative Predictive Value; LR +, likelihood ratio of a positive test; LR -, likelihood ratio of a negative test; No., number; CI, confidence interval

© 2024 Gitaari M et al. *JAMA Network Open*.

eTable 26. True and False Positive and Negative Rates for Each Cutoff for PC-PTSD-5 for Sample With PPCS Present (n = 158)

Cut-Off	TP	FP	TN	FN
≥ *1*	31	58	61	8
≥ *2*	29	47	72	10
≥ *3*	27	31	88	12
≥ *4*	23	20	99	16
≥ *5*	14	8	111	25

Abbreviations: PPV, Positive Predictive Value; NPV, Negative Predictive Value

eTable 27. Diagnostic Accuracy of PC-PTSD-5 for Sample With PPCS Absent (n = 341)

Cut-Off	No. with PTSD (%)	Sensitivity (95%CI)	Specificity (95%CI)	PPV (95%CI)	NPV (95%CI)	LR + (95%CI)	LR- (95%CI)
≥ *1*	6 (1.8)	.67 (0.30-0.93)	.74 (0.69-0.78)	.06 (0.02-0.14)	.99 (0.97-1.00)	2.54 (1.55-4.18)	.45 (0.18-1.14)
≥ *2*	6 (1.8)	.67 (0.30-0.93)	.84 (0.80-0.88)	.10 (0.04-0.21)	.99 (0.97-1.00)	4.18 (2.47-7.05)	.40 (0.16-1.00)
≥ *3*	5 (1.5)	.56 (0.21-0.86)	.92 (0.89-0.95)	.16 (0.05-0.34)	.99 (0.97-1.00)	7.09 (3.55-14.16)	.48 (0.23-1.00)
≥ *4*	4 (1.2)	.44 (0.14-0.79)	.96 (0.93-0.98)	.22 (0.06-0.48)	.98 (0.96-0.99)	10.54 (4.32-25.73)	.58 (0.32-1.04)
≥ *5*	2 (0.6)	.22 (0.03-0.60)	.98 (0.96-0.99)	.22 (0.03-0.60)	.98 (0.96-0.99)	10.54 (2.53-43.83)	.79 (0.56-1.13)

Abbreviations: PTSD, posttraumatic stress disorder; PPV, Positive Predictive Value; NPV, Negative Predictive Value; LR +, likelihood ratio of a positive test; LR -, likelihood ratio of a negative test; No., number; CI, confidence interval

eTable 28. True and False Positive and Negative Rates for Each Cutoff for Sample With PPCS Absent (n = 341)

Cut-Off	TP	FP	TN	FN
≥ *1*	6	87	245	3
≥ *2*	6	53	279	3
≥ *3*	5	26	306	4
≥ *4*	4	14	318	5
≥ *5*	2	7	325	7

Abbreviations: PPV, Positive Predictive Value; NPV, Negative Predictive Value

© 2024 Gitaari M et al. *JAMA Network Open*.

eTable 29. Diagnostic Accuracy of GAD-7 to Diagnose PTSD for Overall Sample (N = 499)

Cut-Off	No. with PTSD (%)	Sensitivity (95%CI)	Specificity (95%CI)	PPV (95%CI)	NPV (95%CI)	LR + (95%CI)	LR- (95%CI)
≥ *6*	44 (8.8)	.92 (0.80-0.98)	.63 (0.58-0.67)	.21 (0.15-0.27)	.99 (0.96-1.00)	2.54 (2.11-2.83)	.13 (0.05-0.34)
≥ *7*	40 (8.0)	.83 (0.70-0.93)	.70 (0.65-0.74)	.23 (0.17-0.29)	.98 (0.95-0.99)	2.74 (2.27-3.31)	.24 (0.13-0.45)
≥ *8*	37 (7.4)	.77 (0.63-0.88)	.76 (0.72-0.80)	.26 (0.19-0.33)	.97 (0.95-0.98)	3.22 (2.57-4.03)	.30 (0.18-0.51)
≥ *9*	34 (6.8)	.71 (0.56-0.83)	.79 (0.75-0.82)	.26 (0.19-0.35)	.96 (0.94-0.98)	3.33 (2.58-4.29)	.37 (0.24-0.58)
≥ *10*	32 (6.4)	.67 (0.52-0.80)	.81 (0.77-0.85)	.27 (0.20-0.36)	.96 (0.93-0.98)	3.54 (2.68-4.67)	041 (0.27-0.61)
≥ *11*	30 (6.0)	.63 (0.47-0.76)	.84 (0.80-0.87)	.29 (0.21-0.39)	.95 (0.93-0.97)	3.86 (2.85-5.23)	.45 (0.31-0.65)
≥ *12*	29 (5.8)	.60 (0.45-0.74)	.86 (0.83-0.89)	.32 (0.22-0.42)	.95 (0.93-0.97)	4.39 (3.17-6.08)	.46 (0.32-0.65)

Abbreviations: PTSD, posttraumatic stress disorder; PPV, Positive Predictive Value; NPV, Negative Predictive Value; LR +, likelihood ratio of a positive test; LR -, likelihood ratio of a negative test; No., number; CI, confidence interval

eTable 30. True and False Positive and Negative Rates for Each Cutoff for GAD-7 to Diagnose PTSD for Overall Sample (N = 499)

Cut-Off	TP	FP	TN	FN
≥ *6*	44	169	282	4
≥ *7*	40	137	314	8
≥ *8*	37	108	343	11
≥ *9*	34	96	355	14
≥ *10*	32	85	366	16
≥ *11*	30	73	378	18
≥ *12*	29	62	389	19

Abbreviations: TP, true positive; FP, false positive; TN, true negative; FN, false negative

eTable 31. Multivariable Regression Model

	Odds Ratio[a]	Lower 95%CI	Upper 95%CI	R2
PC-PTSD-5	2.63	1.79	3.84	N/A
GAD-7	3.15	1.93	5.15	N/A
Overall Model				0.36

Abbreviations: GAD-7, Generalised Anxiety Disorder-7; PC-PTSD-5, Primary Care-PTSD-5; CI, confidence interval; N/A, not applicable
[a]*Odds Ratios scaled to correspond to 25:75 percentile of continuous predictors.*

© 2024 Gitaari M et al. *JAMA Network Open*.

Table 32. AUC Comparison for Each Screening Questionnaire in the Overall, PPCS Present, and PPCS Absent Samples

**Measure**	**Population**	**AUC**	**Lower 95% CI**	**Upper 95% CI**
PHQ-9	Overall (N = 499)	0.911	0.878	0.943
	PPCS + (*n* = 158)	0.782	0.711	0.855
	PPCS – (*n* = 341)	0.829	0.691	0.967
GAD-7 (diagnosing at least one anxiety disorder)	Overall (N = 499)	0.847	0.810	0.883
	PPCS + (*n* = 158)	0.751	0.675	0.827
	PPCS – (*n* = 341)	0.835	0.780	0.891
PC-PTSD-5	Overall (N = 499)	0.798	0.723	0.873
	PPCS + (*n* = 158)	0.747	0.653	0.840
	PPCS – (*n* = 341)	0.760	0.567	0.954

Abbreviations: AUC, area under the curve; CI, confidence interval; PPCS +, persistent postconcussive symptoms present; PPCS -, persistent postconcussive symptoms absent; PHQ-9, Patient Health Questionnaire-9; GAD-7, Generalised Anxiety Disorder-7; PC-PTSD-5, Primary Care-Posttraumatic Stress Disorder-5

© 2024 Gitaari M et al. *JAMA Network Open*.

eReferences

1. Pozzato I, Cameron ID, Meares S, et al. A surveillance study to determine the accuracy of mild traumatic brain injury diagnosis in an emergency department: protocol for a retrospective cohort study. *BMJ Open*. 2017;7(8):e016222. doi:10.1136/bmjopen-2017-016222

2. Pozzato I, Meares S, Kifley A, et al. Challenges in the acute identification of mild traumatic brain injuries: results from an emergency department surveillance study. *BMJ Open*. 2020;10(2):e034494. doi:10.1136/bmjopen-2019-034494

3. Kroenke K, Spitzer RL, Williams JBW. The PHQ-9. *J Gen Intern Med*. 2001;16(9):606-613. doi:10.1046/j.1525-1497.2001.016009606.x

4. Fann JR, Bombardier CH, Dikmen S, et al. Validity of the Patient Health Questionnaire-9 in assessing depression following traumatic brain injury. *J Head Trauma Rehabil*. 2005;20(6):501-511. doi:10.1097/00001199-200511000-00003

5. Donders J, Pendery A. Clinical Utility of the Patient Health Questionnaire-9 in the Assessment of Major Depression After Broad-Spectrum Traumatic Brain Injury. *Arch Phys Med Rehabil*. 2017;98(12):2514-2519. doi:10.1016/j.apmr.2017.05.019

6. Zachar-Tirado CN, Donders J. Clinical utility of the GAD-7 in identifying anxiety disorders after traumatic brain injury. *Brain Inj*. 2021;35(6):655-660. doi:10.1080/02699052.2021.1895315

7. Spitzer RL, Kroenke K, Williams JBW, Löwe B. A Brief Measure for Assessing Generalized Anxiety Disorder: The GAD-7. *Arch Intern Med 1960*. 2006;166(10):1092-1097. doi:10.1001/archinte.166.10.1092

8. Prins A, Bovin MJ, Smolenski DJ, et al. The Primary Care PTSD Screen for DSM-5 (PC-PTSD-5): Development and Evaluation Within a Veteran Primary Care Sample. *J Gen Intern Med JGIM*. 2016;31(10):1206-1211. doi:10.1007/s11606-016-3703-5

9. Bovin MJ, Kimerling R, Weathers FW, et al. Diagnostic Accuracy and Acceptability of the Primary Care Posttraumatic Stress Disorder Screen for the Diagnostic and Statistical Manual of Mental Disorders (Fifth Edition) Among US Veterans. *JAMA Netw Open*. 2021;4(2):e2036733. doi:10.1001/jamanetworkopen.2020.36733

10. Williamson MLC, Stickley MM, Armstrong TW, Jackson K, Console K. Diagnostic accuracy of the Primary Care PTSD Screen for DSM-5 (PC-PTSD-5) within a civilian primary care sample. *J Clin Psychol*. 2022;78(11):2299-2308. doi:10.1002/jclp.23405

© 2024 Gitaari M et al. *JAMA Network Open*.
