# Supplementary material for: Diagnostic Accuracy of Mental Health Screening Tools After Mild Traumatic Brain Injury
Source: JAMA Netw Open. 2024 Jul 23;7(7):e2424076. doi: 10.1001/jamanetworkopen.2024.24076 (PMC11267412; doi:10.1001/jamanetworkopen.2024.24076)
Supplement: Supplement 2. — Data Sharing Statement [file jamanetwopen-e2424076-s002.pdf]

Data Sharing Statement

Gitaari. Diagnostic Accuracy of Mental Health Screening Tools After Mild Traumatic Brain Injury. *JAMA Netw Open*. Published July 23, 2024. doi:10.1001/jamanetworkopen.2024.24076

Data
Data available: Yes
Data types: Deidentified participant data
How to access data: noah.silverberg@ubc.ca
When available: With publication

Supporting Documents
Document types: None

Additional Information
Who can access the data: Researchers whose proposed use of the data has been approved
Types of analyses: For a specified purpose
Mechanisms of data availability: With a signed data access agreement
